# Supplementary biomarker testing in molecular tumor boards increases actionable therapy recommendations: a prospective real-world study of 658 patients

**DOI:** 10.1186/s12916-026-04636-y

**Published:** 2026-01-14

**Authors:** Alexander Scheiter, Simon Mellin, Felix Keil, Johannes Meier, Daniel Heudobler, Christina Brummer, Sabine Einhell, Benjamin Zwicker, Elena Wutzlhofer, Frederik Hierl, Sophie Klemm, Elena Lüftl, Tom Schneider, Markus Perl, Margit Klier-Richter, Alexander Immel, Till Kaltofen, Matthias Grube, Elisabeth Bumes, Stephan Seitz, Christian Schulz, Sebastian Haferkamp, Konstantin Drexler, Anja Troeger, Felix Steger, Sophie Schlosser-Hupf, Hauke Christian Tews, Arne Kandulski, Kristina Wohlfart, Ramona Erber, Ines Schönbuchner, Davor Lessel, Marco J. Schnabel, Anja M. Sedlmeier, Monika Klinkhammer-Schalke, Julia Maurer, Diego F. Calvisi, Tobias Pukrop, Ulrich Kaiser, Daniela Hirsch, Wolfgang Dietmaier, Matthias Evert, Florian Lüke, Kirsten Utpatel

**Affiliations:** 1 Institute of Pathology, University of Regensburg, 93053 Regensburg, Germany; 2 Bavarian Center for Cancer Research / BZKF, Bavaria, Germany; 3 Center for Translational Oncology, University Hospital Regensburg, Regensburg, Germany; 4 Department of Internal Medicine III, Hematology and Oncology, University Hospital Regensburg, 93053 Regensburg, Germany; 5 Department of Surgery, University Hospital Regensburg, 93053 Regensburg, Germany; 6 Department of Neurology and Wilhelm Sander-NeuroOncology Unit, University Hospital Regensburg, 93053 Regensburg, Germany; 7 Department of Gynecology and Obstetrics, University Medical Centre Regensburg, 93053 Regensburg, Germany; 8 Department of Pneumology, University Hospital Regensburg, 93053 Regensburg, Germany; 9 Department of Dermatology, University Hospital Regensburg, 93053 Regensburg, Germany; 10 Department of Pediatric Hematology, Oncology and Stem Cell Transplantation, University Hospital of Regensburg, 93053 Regensburg, Germany; 11 Department of Radiotherapy, Regensburg University Medical Center, 93053 Regensburg, Germany; 12 Department of Internal Medicine I, University Hospital Regensburg, 93053 Regensburg, Germany; 13 Department of Clinical Human Genetics, University Hospital Regensburg, 93053 Regensburg, Germany; 14 Department of Human Genetics, University Regensburg, 93053 Regensburg, Germany; 15 Department of Urology, Caritas St. Josef Medical Center, University of Regensburg, 93053 Regensburg, Germany; 16 Tumour Center - Institute for Quality Management and Health Services Research, University of Regensburg, 93053 Regensburg, Germany; 17 University Cancer Center Regensburg, University Hospital Regensburg, 93053 Regensburg, Germany; 18 Fraunhofer-Institut Für Toxikologie und Experimentelle Medizin ITEM-R, Abteilung Für Personalisierte Onkologie, 93053 Regensburg, Germany; 19 MVZ Dr. Vehling-Kaiser GmbH, Landshut, Germany

**Keywords:** Precision oncology, Molecular tumor board (MTB), Biomarkers, Next-generation sequencing (NGS), Homologous recombination deficiency (HRD), Antibody–drug conjugates (ADC), Microsatellite instability (MSI), Tumor mutational burden (TMB), HER2-low, PD-L1

## Abstract

**Background:**

Molecular tumor boards (MTBs) are essential for selecting therapies for patients with rare and advanced cancers. We hypothesized that integrating biomarkers beyond targeted DNA/RNA next-generation sequencing (NGS) could increase actionable findings. Human epidermal growth factor receptor 2 (HER2)-low status has emerged as a critical biomarker in breast cancer, with potential relevance across other tumor types. Homologous recombination deficiency (HRD) is pivotal for the application of Poly(ADP-Ribose)-Polymerase (PARP) inhibitors in ovarian and breast cancer, although its role in other malignancies remains unclear. Antibody–drug conjugates (ADCs) are expanding precision oncology, with promising biomarkers like Trop-2, Nectin-4, and folate receptor alpha (FRα) showing potential across multiple tumor entities.

**Methods:**

Tumors were analyzed using the TSO500® panel, enabling tumor mutational burden (TMB) readout. HER2 status was assessed via immunohistochemistry (IHC) and fluorescence in situ hybridization (FISH), alongside antibody–drug conjugate (ADC) IHC, microsatellite instability (MSI) polymerase chain reaction (PCR), mismatch repair (MMR) IHC, programmed death-ligand 1 (PD-L1) IHC, and HRD analysis. Cases were discussed weekly, and outcomes were systematically tracked. Data analysis evaluated the benefit of additional biomarker assessments.

**Results:**

Among 658 patients, 329 received therapy recommendations, 182 based on supplementary biomarker analyses. One hundred recommendations were implemented, with 37% attributed to supplementary diagnostics. Among 64 response-evaluable patients, the clinical benefit rate (complete response + partial response + stable disease) was 45.3%. HER2-low status notably expanded targeted therapy options across tumor types, with similar implementation rates for HER2-low and HER2-amplified tumors. HRD analysis refined stratification in tumors with mutations in homologous recombination repair (HRR) genes beyond BRCA1/2, including PALB2, ATM, and CHEK2. ADC IHC supported 20 recommendations and two therapy implementations.

**Conclusions:**

The integration of additional biomarker assessments into MTB workflows enhances precision oncology by expanding the pool of patients eligible for targeted therapies.

**Supplementary Information:**

The online version contains supplementary material available at 10.1186/s12916-026-04636-y.

## Background

Precision oncology, the tailored application of therapeutic strategies based on molecular and genetic profiling, has revolutionized cancer treatment [1]. Molecular tumor boards (MTBs) are central to this approach, providing a multidisciplinary framework for the integration of genomic, sometimes transcriptomic, and immunohistochemical data to guide individualized therapy decisions [2]. While conventional genetic panel diagnostics have become the cornerstone of MTB decision-making, the addition of supplementary diagnostic methods holds the potential to further enhance the utility of MTBs beyond the upcoming integration of exome and whole-genome sequencing.

BRCA1/2 mutations and homologous recombination deficiency (HRD), for example, is a well-established bio-marker for the application of PARP inhibitors in ovarian [3] and breast cancer [4]; however, its utility in other malignancies is less defined, as is the relevance of mutations in other homologous recombination repair (HRR) genes such as PALB2, etc. Similarly, the advent of antibody–drug conjugates (ADCs) has introduced a new dimension to precision oncology, with bio-markers such as Trop-2, Nectin-4, and FRα showing promise across an increasing varity of tumor types [5–8]. Furthermore, the assessment of microsatellite instability (MSI) through PCR-based methods and IHC for mismatch repair (MMR) proteins represent key approaches to identification of potential candidates for immune checkpoint inhibitors (ICI), particularly in gastrointestinal and endometrial cancers [9]. However, the reliability of MSI scores obtained from sequencing panels, particularly regarding the definition of cut-off values, remains a topic of active discussion [10]. Another well-recognized bio-marker for immuno-oncological therapies is PD-L1, which has proven its utility in various cancer types [11–13] which was consequently also integrated into our diagnostic workflow.

Moreover, HER2 expression, evaluated via IHC and FISH, has expanded beyond HER2-positive, i.e., HER2-amplified tumors to include HER2-low as a relevant subgroup. The introduction of Trastuzumab Deruxtecan (T-DXd) has shown efficacy in HER2-low breast cancer [14], with emerging data suggesting potential benefits in other malignancies [15].

Studies evaluating the impact of supplemental diagnostics in the MTB setting are scarce [16, 17]. While the incorporation of biomarkers such as HRD analysis and ADC-specific IHC into MTBs seams promising, their incremental value in expanding actionable findings and influencing clinical recommendations has yet to be comprehensively evaluated.

To address this question, we conducted an extensive analysis of 658 patients presented at our institutional MTB from January 2022 to November 2024, focusing on the incorporation of supplemental diagnostic modalities alongside standard panel sequencing. The study aimed to evaluate whether incorporating HRD analysis, HER2 IHC and FISH, ADC IHC, MSI-PCR, and PD-L1 IHC would improve the number and quality of actionable recommendations. Additionally, the study sought to assess the feasibility of integrating these diagnostics into routine MTB workflows. Feasibility was assessed in terms of the proportion of patients for whom these assays generated additional recommendations, the rate of implementation of such recommendations in clinical practice, and the associated patient outcomes.

## Methods

### Study design and patient population

This prospective registry study was conducted at the University Hospital Regensburg under the approval of the institutional ethics committee (Protocol Number: 20–1682-101). Patient recruitment began in 2019, with the current evaluation focusing on patients included between 2022 and 2024. The earlier cohort (2019–2021) has already been reported previously [17], and the present study was resticted to 2022–2024 because supplementary biomarker testing was systematically implemented into the MTB workflow only from 2022 onwards. The study targeted individuals with advanced malignancies meeting specific inclusion criteria. These criteria included (1) exhaustion or near exhaustion of guideline-based therapies, (2) initiation of the last line of therapy with limited expected efficacy, (3) rare tumor entities without standard-of-care treatment options, and (4) sufficient life expectancy, estimated at a minimum of 6 months, to enable sufficient time for molecular testing and individual treatment authorization processes.

Patients also needed to provide informed consent, have tissue or DNA samples readily available for analysis, and demonstrate openness to experimental and off-label therapies, which was checked during pre-MTB counseling by the treating physician and recorded in the MTB case form as part of the inclusion process. Procedural requirements included written consent, referral from the organ-specific tumor board for extended molecular analysis, a comprehensive medical record for therapy documentation, and an assessment of the defined inclusion criteria.

Molecular findings were first reported to the treating physician as molecular pathology report without therapeutic interpretation. Prior to the MTB, alterations were jointly annotated by pathologists and clinicians based on literature review from our institutional database. The annotated reports were then shared with the treating physicians, particularly with designated ‘bridgeheads’ responsible for preparing and presenting cases at the MTB.

The MTB convened weekly and involved a core team composed of a clinical geneticist, pathologist, medical oncologist, molecular pathologist/biologist, and a rotating affiliated clinician collaboratively evaluating the cases. The Centers of Personalized Medicine (ZPM) scheme was used to assign evidence levels [18], which is based on the MD Anderson Cancer Center classification and stratifies biomarkers according to their level of clinical and scientific validation. Tier m1 and m2 categorize biomarkers based on clinical evidence within the same tumor type (m1) or a different tumor type (m2), ranging from prospective studies (A), retrospective studies (B), and case reports (C). Tier m3 and m4 refer to preclinical evidence (m3) and theoretical biological rationale (m4) without supporting biomarker-stratified clinical data. Initially, the m4 rating was only occasionally assigned. Through the subsequent clinical reassessment, cases with with a clear underlying biological rationale for treatment recommendations were assigned this category retrospectively, including recommendations of clinical trials with an underlying biomarker dependency. Recommendations for genetic counseling were made based on the evaluation of the molecular test results by a geneticist. Exceptions to this workflow were predominantly lung cancer cases, where routine in-house panel diagnostics had yielded uncommon or ambiguous alterations. For these “consult” cases only ADC-IHC was supplemented to the already available panel diagnostics. Moreover, within the period of evaluation, cholangiocarcinoma (CCA) obtained several newly approved and molecularly guided treatment options, such as the use of FGFR inhibitors based on FGFR2-fusions [19]. Nonetheless, CCA patients were continuously enrolled directly with the start of first-line therapies to allow for a cost-efficient testing without the need for repeated genetic evaluations in due course. Moreover, the recently updated German guidelines reinforce the strategy of early referral of CCA patients to the MTB after failure of first-line therapy [20].

### Analysis methods by tumor entities

The standard analytical framework included the TruSight Oncology 500® panel (TSO500®; Illumina Inc., San Diego, CA, USA), whose diagnostic reliability in clinical practice has just recently been confirmed [21], PD-L1 IHC, MMR protein analysis via IHC for PMS2 and MSH6 (we shifted from an initial assessment of MSH2, MSH6, PMS2, and MLH1 to only these two markers out of economic considerations). HER2 diagnostics using IHC, supplemented by fluorescence in situ hybridization (FISH) when necessary, were also standard. Generally, the Rüschoff score was used in analogy to gastric cancer [22]. Cases with a score of 2 + were sent for FISH analysis taking into consideration that other entities have a less established diagnostic framework for HER2 IHC diagnostics.

For certain tumor entities, additional assays were incorporated, such as ADC-targeted IHCs introduced in early 2024 and HRD assessments. Before 2024, these supplementary parameters were not routinely evaluated. An overview of the standard allocation of diagnostics by entity in our MTB is presented in Additional file 1: Table S1. All additional biomarkers beyond mutation and gene fusion analysis by targeted DNA/RNA-NGS were considered as “supplementary” diagnostics.

### Clinical reassessments

Patient data and therapeutic recommendations were systematically documented in standardized MTB case documents. We extracted data from these documents using a custom Python script, incorporating various modules for processing. Different data tables (e.g., mutations, fusions, PD-L1) were identified, structured, and stored in an SQLite database (Hipp, Wyrick & Company, Inc., Charlotte, North Carolina, USA), requiring adaptations for consistent parsing. Free text outside tables was generally ignored, except for specific cases such as searching for references to genetic counseling in recommendation texts. Implementations of recommendations was determined through systematic review of follow-up documentation, including treatment protocols, discharge letters, and physician notes. Only therapies that could be clearly matched to the MTB recommentation were classified as implemented. In cases where no matching therapy was documented, recommendations were considered not implemented. Clinical follow-up data were collected at 1, 3, 6, 12, 18, and 24 months post-therapy by sending standardized follow-up documentation files to the treating physicians. Challenges in follow-up compliance led to supplementary approaches, including contacting patients, physicians, and local cancer registries, and reviewing publicly available death notices. For survival analysis, censoring was applied at the last documented contact date if no subsequent data were available. The final data cut-off was August 16, 2025.

Therapeutic responses were categorized based on clinical and radiological assessments as progressive disease (PD), stable disease (SD), partial response (PR), mixed response (MR), or complete response (CR). Progression-free survival (PFS) was defined as the time from treatment initiation to disease progression. Progression events were determined based on radiological criteria. If no progression event was recorded, the end of therapy or the end of the observation period was considered a censored event. PFS was calculated for both pre- and post-MTB therapy lines, with intra-patient benefit assessed by PFS ratio (PFS2/PFS1), where values above 1.3 were considered indicative of therapeutic benefit [23].

For comparison purposes, we also analyzed treatments implemented after the molecular tumor board, which do not represent targeted therapies and were independent of MTB recommendations with the same methodology as we analyzed MTB therapies. This group is referred to as Non-MTB therapy group and comprises patients irrespective of given MTB recommendations. In addition, we defined a no additional therapy cohort comprising patients who, following MTB discussion, either continued their existing treatment without modification or received no subsequent systemic therapy. This group again includes both patients with and without MTB recommendations. PFS could not be assessed in patients who did not receive further systemic therapy, as disease progression was not systematically evaluated in this group.

### HRD analyses

HRD assessments were performed using two distinct panels: the TSO-500 with integrated HRD analysis (Illumina, cut-off for positivity: GIS ≥ 42), which has proven concordance with the FDA-approved companion diagnostics MyChoice®CDx PLUS assay (Myriad assay) [24, 25], and the QIAseq Targeted DNA Custom Panel (96) (QIAGEN N.V., Venlo, Netherlands, cut-off for positivity: GIS ≥ 56). The TSO-500 HRD panel was generally preferred, while the Qiagen panel was employed for cases with prior TSO-500 analysis lacking HRD evaluation or when HRD analysis hat already been performed in consiliary cases.

### ADC IHC

ADC-related IHC analyses included the markers Nectin-4, Trop-2, Tissue Factor (TF), FRα, and Claudin18.2. It should be noted that the drug Zolbetuximab targeting Claudin18.2 is not an ADC by definition. Instead, it elicits antibody-dependent cell- and complement-dependent cytotoxicity to eliminate cancer cells [26]. Nonetheless, it is subsumed as ADC in this publication. The IHC assessment of the abovementioned targets was implemented starting in 2024 and adhered to the H-score [27], which quantifies the staining intensity (0, 1 + , 2 + , or 3 +) of positive tumor cells as a percentage of the total tumor cell count. The H-score ranges from 0 (all tumor cells negative) to 300 (all tumor cells strongly positive). Examples of H-Score assessments for various ADC antibodies are presented in Additional file 1: Fig. S1_1 and Fig. S1_2. At least one board-certified pathologist reviewed each sample, utilizing standardized positive controls. Additional file 1: Table S2 lists the antibodies and technical parameters. Until August 2024, the FRα antibody clone from Novocastra was used; thereafter, the one from Ventana was adopted.

The choice of ADC-IHC was based on the following detailed rationale: TROP-2 is frequently expressed in urothelial carcinoma and breast cancer, particularly in triple-negative breast cancer (TNBC) [28]. In the ASCENT study, a biomarker analysis of TROP-2 was conducted [29]. Here, 80% of patients with metastatic TNBC had high or medium TROP-2 expression. In this subgroup, Sacituzumab Govitecan showed improved survival outcomes and response rates compared to treatment of physician’s choice. Due to the small number of patients with low TROP-2 expression, no definitive conclusions could be drawn, though a trend toward lower response rates and overall survival was observed.

Regarding urothelial carcinoma, in cohorts 1–3 of the Phase II TROPHY-U-01 study (mUC after platinum-based chemotherapy and checkpoint inhibitors), tumor samples were collected from 192 patients, and evaluable TROP-2 data were available for 146 patients (76%). The median H-score was 215 (scale 0–300), with a median percentage of membrane-stained cells of 91% (range 80–98%). TROP-2 was expressed in 98% of patients, and SG demonstrated efficacy across all levels of expression—objective response rate (ORR), progression-free survival (PFS), and overall survival (OS) were similar across the cohort [30, 31]. In summary SG showed somewhat reduced activity in low TROP-2-expressing TNBC, while efficacy in urothelial carcinoma was similar across all expression groups. At present, TROP-2 is not yet a validated predictive biomarker. Nevertheless, we have chosen to assess TROP-2 expression in certain tumor entities, such as breast cancer, urothelial carcinoma, and cancer of unknown primary (CUP), in order to evaluate whether expression levels are indeed as high as reported in clinical trials.

In the Phase I trial of enfortumab vedotin (EV) in urothelial carcinoma, evaluation of Nectin-4 expression by immunohistochemistry (IHC) was initially required for enrollment. For each patient, Nectin-4 expression was quantified using a histochemical scoring system based on staining intensity multiplied by the percentage of positively stained cells (H-score, range 0–300). Nectin-4 was detected in 97% of patients, with a median H-score of 290 (range 14–300). Due to the consistently high expression, positive Nectin-4 testing was later removed as an eligibility criterion [32].

In a larger retrospective study involving 137 matched primary tumor-metastasis pairs and a separate EV-treated cohort, it was shown that Nectin-4 expression frequently decreased in metastases—in 39% of metastases, expression was absent or markedly reduced. Weak or absent membranous expression (H-score 0–99) was associated with shorter PFS following EV treatment (log-rank *p* < 0.001). The authors concluded that low or absent expression may predict resistance to EV, supporting the strategy of biopsying metastases and testing for Nectin-4 expression before initiating EV therapy [33]. High Nectin-4 expression has also been described in other entities such as lung cancer and squamous cell carcinomas, which is why we tested these entities alongside CUP.

Tissue factor (TF) is also expressed in a range of tumor types, including cervical cancer, non-small cell lung cancer (NSCLC), and head and neck squamous cell carcinoma (HNSCC). Tisotumab Vedotin, an antibody–drug conjugate targeting TF, was approved based on the results of the randomized Phase III innovaTV 301 study (NCT04697628) in metastatic cervical cancer [34]. Unfortunately, no biomarker analyses were published for this study. However, a generally high TF expression is assumed for this tumor entity. Given the limited available data, we decided to restrict TF testing to squamous cell carcinomas of the head and neck.

### Statistical analyses and software

Descriptive statistics were conducted using Microsoft Excel (Version 16, Microsoft Corporation, Redmond, WA). GraphPad Prism version 9 (Graphpad Software, LLC, San Diego, CA, USA) was utilized for survival analyses. Kaplan–Meier curves and Log-rank tests were used to compare OS between the groups with no therapy and MTB therapy.

### Genomic, FISH, MSI, and general IHC protocol

The exact procedure for genomic analyses, PCR fragment sizing MSI analysis, FISH, and the general IHC procedure including HER2-IHC (while the specifical parameters for ADC-IHC are reported here) has been published previously by us and was continuously used in an unaltered manner [17]. MSI testing was defined as MMR IHC and/or MSI PCR.

MSI status was assessed using the Illumina TruSight Oncology 500 (TSO500) panel. A cutoff of ≥ 10% unstable loci was applied, reflecting established consensus among German molecular pathology expert groups for this assay. Although this specific threshold has not yet been formally published, it has been validated in our laboratory through benchmarking and inter-laboratory exchange. This threshold has also been employed by Pestinger et al. [35]. To ensure accuracy, all cases with ≥ 10% unstable loci also underwent PCR and fragment-length analysis, which served as the gold standard for final MSI classification. This approach was chosen to maximize sensitivity and to avoid missing borderline cases, for example in samples with low tumor cell content. Recommendations were never made on TSO500 MSI analysis alone, but had to be confirmed by either MMR IHC or PCR and fragment-length analysis (when normal tissue was available for comparison).

In rare instances, androgen receptor or estrogen receptor IHC was carried out and used as rationale for therapy recommendation.

## Results

### *Patient population and molecular genetic analyses (*Fig. 1*, *Table 1*)*

**Fig. 1:**
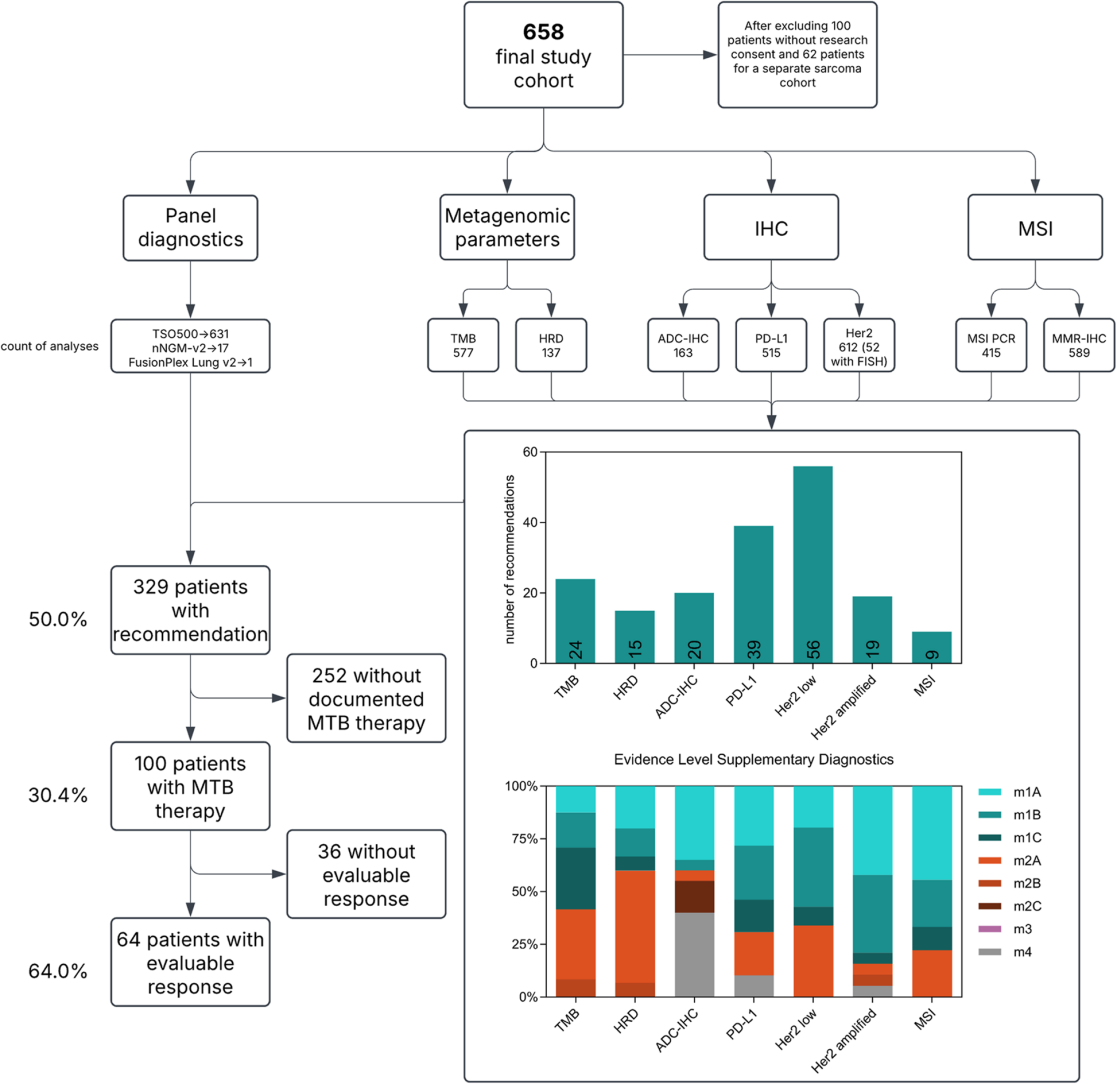
Study overview and supplementary analyses. The flow chart illustrates the diagnostic workflow focusing on supplementary diagnostics alongside the panel diagnostics, as well as the resulting recommendations and their clinical follow-up. Percentages in the flow chart are relative to the previous hierarchical level. The included bar chart (top) illustrates the number of recommendations provided for each diagnostic category. The stacked bar plot (bottom) depicts the distribution of evidence levels (m1A–m4) across different supplementary diagnostic parameters. One hundred eighty-two patients received therapy recommendations based on the defined supplementary diagnostic methods, while the remainder of patients (147) had recommendations derived only from next-generation sequencing analyses (with rare exceptions of androgen receptor and estrogen receptor immunohistochemistry)

**Table 1:** Patient characteristics

	All patients	MTB therapy	Non-MTB therapy	No additional therapy
Population	658	100	89	469
Mean age, standard deviation (years)	60.0 ± 13.2	58.3 ± 12.9	58.2 ± 15.1	60.7 ± 12.9
Sex				
Male (*n*, %)	389 (59.1%)	55 (55.0%)	55 (61.8%)	279 (59.5%)
Female (*n*, %)	269 (40.9%)	45 (45.0%)	34 (38.2%)	190 (40.5%)
ECOG PS (total, remaining unknown)	370	38	59	273
0	170	21	38	111
1	155	15	15	125
2	31	1	6	24
3	12	1	0	11
4	1	0	0	1
5	1	0	0	1
Stage of cancer				
I	2 (0.4%)	0	1 (1.6%)	1 (0.2%)
II	13 (2.7%)	5 (6.7%)	1 (1.6%)	7 (1.5%)
III	28 (5.9%)	4 (5.3%)	4 (6.3%)	20 (4.3%)
IV	432 (90.9%)	66 (88%)	57 (90.5%)	309 (65.9%)
Total (remaining unknown)	475	75	63	337
MTB Recommendations (*n*, %)	329 (50%)	100 (100%)	39 (43.8%)	190 (40.5%)
Evidence level primary recommendation (*n*, %)				
m1A	88 (26.8%)	34 (34%)	9 (23.1%)	45 (9.6%)
m1B	70 (21.3%)	21 (21%)	8 (20.5%)	41 (8.7%)
m1C	45 (13.7%)	19 (19%)	4 (10.3%)	22 (4.7%)
m2A	37 (11.3%)	9 (9%)	8 (20.5%)	20 (4.3%)
m2B	6 (1.8%)	6 (6%)	1 (2.6%)	0
m2C	12 (3.7%)	2 (2%)	1 (2.6%)	9 (1.9%)
m3	4 (1.2%)	3 (3%)	0	1 (0.2%)
m4	2 (0.6%)	0	0	2 (0.4%)
Clinical trial	64 (19.5%)	6 (6%)	8 (20.5%)	50 (10.7%)
human genetic counseling	146 (22.2%)	32 (32%)	20 (22.5%)	94 (20.0%)
Entity (*n*, %)				
CCA	120 (18.2%)	16 (16%)	20 (22.5%)	84 (17.9%)
CRC	78 (11.9%)	9 (9%)	16 (18%)	53 (11.3%)
Pancreatic cancer	56 (8.5%)	5 (5%)	5 (5.6%)	46 (9.8%)
CUP	45 (6.8%)	9 (9%)	9 (10.1%)	27 (5.8%)
Prostate cancer	38 (5.8%)	1 (1%)	3 (3.4%)	34 (7.2%)
Breast cancer	35 (5.3%)	10 (10%)	7 (7.9%)	18 (3.8%)
NSCLC	35 (5.3%)	11 (11%)	6 (6.7%)	18 (3.8%)
Esophagogastric cancer	32 (4.9%)	5 (5%)	1 (1.1%)	26 (5.5%)
Bladder cancer	28 (4.3%)	1 (1%)	2 (2.2%)	25 (5.3%)
Head and neck cancer	24 (3.7%)	1 (1%)	2 (2.2%)	21 (4.5%)
Ovarian cancer	18 (2.8%)	3 (3%)	1 (1.1%)	14 (3.0%)
Melanoma	17 (2.6%)	5 (5%)	3 (3.4%)	9 (1.9%)
Salivary gland cancer	15 (2.3%)	4 (4%)	2 (2.2%)	9 (1.9%)
Mature T and NK neoplasms	12 (1.8%)	3 (3%)	0	9 (1.9%)
Small bowel cancer	11 (1.7%)	0	1 (1.1%)	10 (2.1%)
Renal cell carcinoma	10 (1.5%)	0	0	10 (2.1%)
HCC	7 (1.1%)	0	0	7 (1.5%)
Penile cancer	7 (1.1%)	1 (1%)	1 (1.1%)	5 (1.1%)
Thyroid cancer	6 (0.9%)	3 (3%)	1 (1.1%)	2 (0.4%)
Appendiceal cancer	5 (0.8%)	0	1 (1.1%)	4 (0.9%)
Bowel cancer	4 (0.6%)	1 (1%)	0	3 (0.6%)
Cervical cancer	4 (0.6%)	0	0	4 (0.9%)
Endometrial cancer	4 (0.6%)	2 (2%)	0	2 (0.4%)
Thymic tumor	4 (0.6%)	0	0	4 (0.9%)
Others (*n* ≤ 3 in all patients)	43 (6.5%)	10 (10%)	8 (9%)	25 (5.3%)

We first describe the study cohort and the scope of molecular and supplementary diagnostics performed. Of the 820 patients initially discussed at our MTB between 2022 and 2024, 100 did not provide research consent and 62 were excluded as part of a separately analyzed sarcoma cohort, leaving 658 patients for inclusion in this study.

The overall study cohort had a median age of 60.0 years (SD ± 13.2), while the MTB-guided therapy subgroup exhibiting a slightly lower median age of 58.3 years (SD ± 12.9). Men comprised 59.1% (*n* = 389) of the cohort. ECOG performance status was reported for 370 patients (56.2%), with most having an ECOG score of 0 (46%) or 1 (41.9%). Among all groups, cancers included were primarily of advanced stage (UICC IV, ~ 90%), while only occasionally lower stage cancers were admitted to the MTB.

Comprehensive molecular diagnostics were performed using targeted sequencing panels in 646 cases (TSO 500: *n* = 631; NNGM v2 and v3.2: *n* = 13) and a fusion panel in two cases (Archer FusionPlex Lung V2). In the remaining 12 cases, sequencing was not conducted for various reasons, including insufficient tumor tissue or an unexpected clinical deterioration of the patient, leading to a transition to best supportive care (BSC).TMB assessment by NGS was available for 577 patients, HRD testing was performed in 137 cases, ADC IHC in 163, PD-L1 status in 515, and HER2 IHC in 612, with 52 cases undergoing an additional FISH analysis. Microsatellite instability (MSI) testing by PCR was performed in 415 cases, and MMR protein IHC in 589.

To characterize the tissue used for biomarker analyses, we assessed the tissue and sample types employed for both IHC and next-generation sequencing (Additional file 1: Fig. S2). The majority of samples were biopsies (*n* = 380), followed by resection specimens (*n* = 221). In a few instances, cytological samples (*n* = 4) or liquid biopsies (*n* = 3) were used for molecular analysis. Regarding tumor origin, the most frequent sample type was distant metastasis (*n* = 262), closely followed by primary tumors (*n* = 248). Additional samples included lymph node metastases (*n* = 44), local recurrences (*n* = 43), and in rare cases, blood samples. In general, we aimed to analyze tissue that was less than 6 months old. Nevertheless, exceptions were made in cases where obtaining fresh or repeat biopsies would have posed a significant risk to the patient. In such situations, older tissue was used (median sample age: 103 days).

Actionable targets that could be identified varied across different diagnostic approaches, with ADC-IHC leading to 20 recommendations (of 163 analyses; 12.3%), HRD analyses to 15 (of 137 analyses; 10.9%), MSI testing to 9 (of 600 analyses, 1.5%), TMB assessment to 24 (of 577 analyses; 4.2%), and PD-L1 expression analysis to 39 (of 515 analyses; 7.6%). HER2 IHC resulted in 75 actionable recommendations (of 612 analyses; 12.3%), of which 19 were based on HER2-amplified and 56 on HER2-low status. The evidence supporting these recommendations varied, with ADC-IHC findings predominantly based on m1A (35%) and m4 (40%) evidence. The evidence was unevenly distributed between the different ADC targets. Claudin 18.2, for instance, achieved m1A-level evidence in 80% (4/5 cases with recommendation) of cases, whereas TF and Trop-2 recommendations were only supported by m4-level evidence. HRD recommendations were largely supported by m2A (53.3%), based on evidence primarily derived from breast and ovarian cancer, while MSI findings had strong tumor-specific evidence, with 44.4% classified as m1A. TMB-based recommendations were distributed across m1C (29.2%) and m2A (33.3%), whereas PD-L1-derived recommendations were most frequently supported by m1A (28.2%), m1B (25.6%) and m2A (20.5%) evidence. Recommendations based on HER2-amplified cases were mainly m1A (42.1%) and m1B (36.8%), while the strength of HER2-low recommendations was more diverse, with m1B (37.5%) and m2A (33.9%) being most common.

From the overall cohort (658 patients), 329 patients (50.0%) received a therapy recommendation based on molecular and additional biomarker results. In 329 patients, no therapy recommendation was issued. The predominant reason was the absence of actionable molecular alterations or prior administration of targeted therapies addressing the identified alterations. In a subset of patients, clinical deterioration, comorbidities limiting treatment options, or patient preference precluded further therapeutic intervention. A total of 182 patients received therapy recommendations based on the defined supplementary diagnostics, while 147 patients received recommendations derived from next-generation sequencing results alone. One hundred patients (30.4%) received MTB-guided therapies. Documented outcomes were available for 64 patients (64%).

### Recommendations across tumor entities and underlying alterations

Next, we report the distribution of recommendations across tumor entities (Additional file 1: Fig. S3). The proportion of cases receiving MTB-guided therapy recommendations varied across tumor entities, reflecting differences in the suitability of molecular diagnostics. CCA (*n* = 120) exhibited a recommendation rate of 56.7%, supported by both tumor-specific and cross-entity biomarker-stratified evidence. Breast cancer (*n* = 35) demonstrated the highest recommendation rate among the 5 most prevalent entities at 77.1%. In contrast, colorectal cancer (CRC) (*n* = 78) had a markedly lower recommendation rate of 23.1%. Pancreatic cancer (*n* = 56) and prostate cancer (*n* = 38) also showed comparatively low recommendation rates (28.6% and 36.8%), due to limited high-evidence biomarkers or available targeted therapies.

Apart from the most prevalent entities, some cancers exhibited particularly high recommendation rates. Thyroid cancer received MTB-guided therapy recommendations in 100% of cases (4/4) as a consequence of the high prevalence of PD-L1 expression. Cervical cancer (3/4, 75%), bladder cancer (19/28, 67.9%), and salivary gland cancer (10/15, 66.7%) also demonstrated a strong alignment between molecular diagnostics and actionable findings.

The distribution of evidence levels further reflected variation between entities. Breast and bladder cancer recommendations were predominantly supported by high-evidence biomarkers (m1A and m1B). Thyroid cancer relied heavily on m1B evidence (83.3%). In contrast, pancreatic cancer and cancer of unknown primary (CUP) depended largely on cross-entity evidence (m2A and m2C).

Differences between the tumor entities were also noted regarding the number of recommendations per case. Salivary gland cancer (80%) and CRC (72.2%) frequently yielded multiple actionable targets. Breast cancer also showed a high proportion of multiple recommendations (59.3%), consistent with its strong molecular subtyping. Conversely, renal cell carcinoma and small bowel cancer exclusively received single recommendations.

Overall, this analysis highlights the strong suitability of molecular diagnostics for CCA, breast cancer, thyroid cancer, and bladder cancer due to their high recommendation rates and robust supporting evidence. Conversely, entities such as pancreatic cancer and CRC may benefit from a different and expanded molecular testing approach, to identify candidates for targeted therapy.

Next, we analyzed the genetic alterations underlying treatment recommendations (Additional file 1: Fig. S4). *KRAS* and *BRAF* alterations supported 23 therapy recommendations each (8.3% each). *BRAF* alterations underlying recommendations included 16 mutations and 7 fusions, whereas *KRAS* changes were exclusively mutations. *PIK3CA* mutations were associated with 17 recommendations (6.1%), followed by *FGFR2* alterations with 16 (11 fusions, 4 mutations, and 1 combined alteration, 5.8%). *EGFR* alterations led to a recommendation in 13 cases (11 mutations, 2 amplifications, 4.7%), along with MET (11 amplifications, 1 fusion, 1 combination, 4.7%). *ALK* alterations were the basis of 12 recommendations (3 fusions, 9 mutations, 4.3%), matching the amount of recommendations based on *IDH1* mutations (12, 4.3%).

In CCA, *KRAS* mutations were the most frequent alterations underlying treatment recommendations (19 recommendations, 28.4%), followed by *FGFR2* (15 recommendations, 22.4%, predominantly fusions), *IDH1* (9 recommendations, 13.4%), and *BAP1* (5 recommendations, 7.5%). Among non-small cell lung cancer (NSCLC) cases, *ALK* and *BRAF* to 4 (12.1%), while *ROS1* alterations (2 fusions, 1 combination of alterations, 9.1%) were less common. In breast cancer, actionable alterations included *PIK3CA* (4 recommendations, 19.0%), *ESR1* (3 recommendations, 14.3%), *PTEN* (3 recommendations, 14.3%), as well as *BRCA1*, *PALB2*, and *FGFR1* (all mutations except one FGFR1 fusion) with 1 recommendation (4.8%) each. Five cases involved recommendations based on individual combinations of biomarkers (23.8%). In CRC, the most frequently detected alterations used as basis for therapeutic recommendation were *BRAF* (4 recommendations, 28.6%), *PIK3CA* (4 recommendations, 28.6%), and *KRAS* (3 recommendations, 21.4%) mutations.

As expected, CUP exhibited a diverse recommendation profile, with *ALK* fusions (3 recommendations, 17.6%) and combination-based targets (3 recommendations, 17.6%) being the most common. Single cases with actionable mutations included *AKT1*, *ATM*, *BRAF*, *BRCA2*, *EGFR*, and *NRAS*, as well as *FGFR3* fusion and a *VHL* (5.9% each) mutation.

### *Detailed evaluation of HER2 low and amplification status (*Fig. 2*)*

**Fig. 2:**
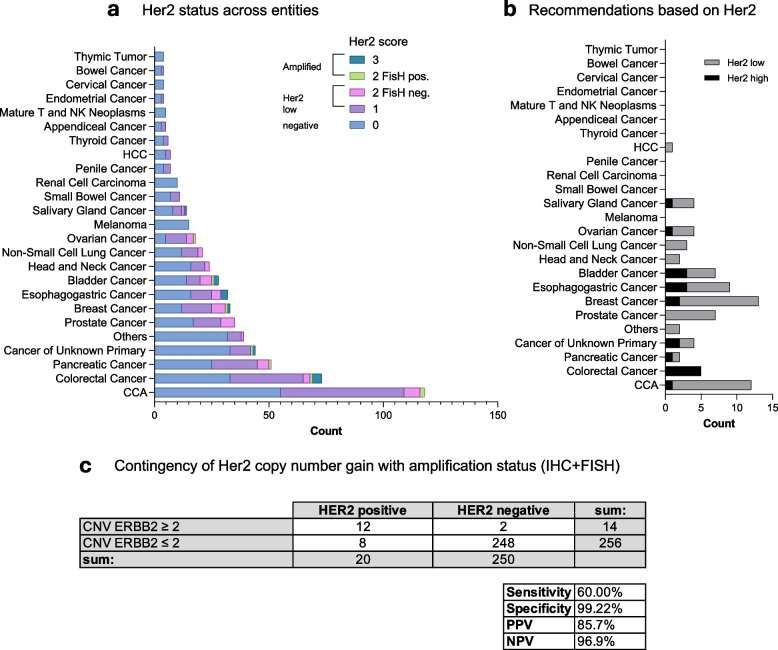
Supplementary diagnostics: HER2. a Distribution of HER2 status across various tumor types. Cases are classified as amplified, low expression, and negative. Tumor types are ranked by frequency of HER2 alterations. Scores are indicated as either DAKO-score (breast cancer) or Rüschoff-Score (other entities). b Comparison of recommendations based on low versus high HER2 expression levels across different tumor types. c The contingency table illustrates HER2-positive and HER2-negative cases based on two independent diagnostic methods (CNV analysis and HER2 IHC + FISH). Sensitivity, specificity, positive predictive value (PPV), and negative predictive value (NPV) are provided

The distribution of HER2 expression varied across different tumor types. In CCA, 55 out of 118 (46.6%) cases were HER2-negative, 61 (51.7%) were classified as HER2-low, and 2 (1.7%) showed amplification. A similar pattern was observed in CRC, with 33 out of 73 (45.2%) cases classified as HER2-negative, 35 (47.9%) as HER2-low, and 5 (of 73, 6.8%) as amplified. In contrast, CUP had a higher proportion of HER2-negative cases (33 of 44, 75%). In breast cancer, 12 out of 33 (36.4%) cases were HER2-negative, 19 (57.6%) were HER2-low, and 1 (6.1%) exhibited amplification. Ovarian cancer had the highest proportion of HER2-low status, observed in 12 out of 18 cases (66.7%). At the same time, it had the lowest HER2-negative rate, with only 5 out of 18 cases (27.8%) classified as negative.

Among HER2-based recommendations, HER2-low status was the primary underlying alteration in CCA (11/12 recommendations, 91.7%), breast cancer (11/13 recommendations, 84.6%), and prostate cancer (7/7 recommendations, 100%). In contrast, CRC recommendations were exclusively based on HER2 amplification (5/5 recommendations). Esophagogastric and bladder cancers had recommendations based on HER2-low status in 66.7% and 57.1% of cases, respectively.

When comparing *HER2 (ERBB2)* copy number variation (CNV) detected by sequencing (a CNV ERBB2 value of ≥ 2 was considered positive) with IHC/FISH results, sequencing demonstrated high specificity (99.2%) but only moderate sensitivity (60%) as well as high positive (85.7%) and negative (96.9%) predictive values. This indicates that CNV assessment is reliable for ruling out HER2 positivity but may miss some amplified cases, probably due to focal amplification or technical limitations due to insufficient tumor cell content. The high positive (85.7%) and negative (96.9%) predictive values confirm that most amplified CNVs correspond to true HER2-positive cases. Given the limited sensitivity of sequencing, IHC and FISH remain essential for accurately classifying HER2 status, particularly in cases where CNV results are inconclusive, sequencing quality is suboptimal or tumor cell content is low. Integrating these methods ensures a comprehensive assessment and supports correct treatment selection.

### *Assessing ADC-IHC diagnostics in the MTB framework (*Fig. 3*)*

**Fig. 3:**
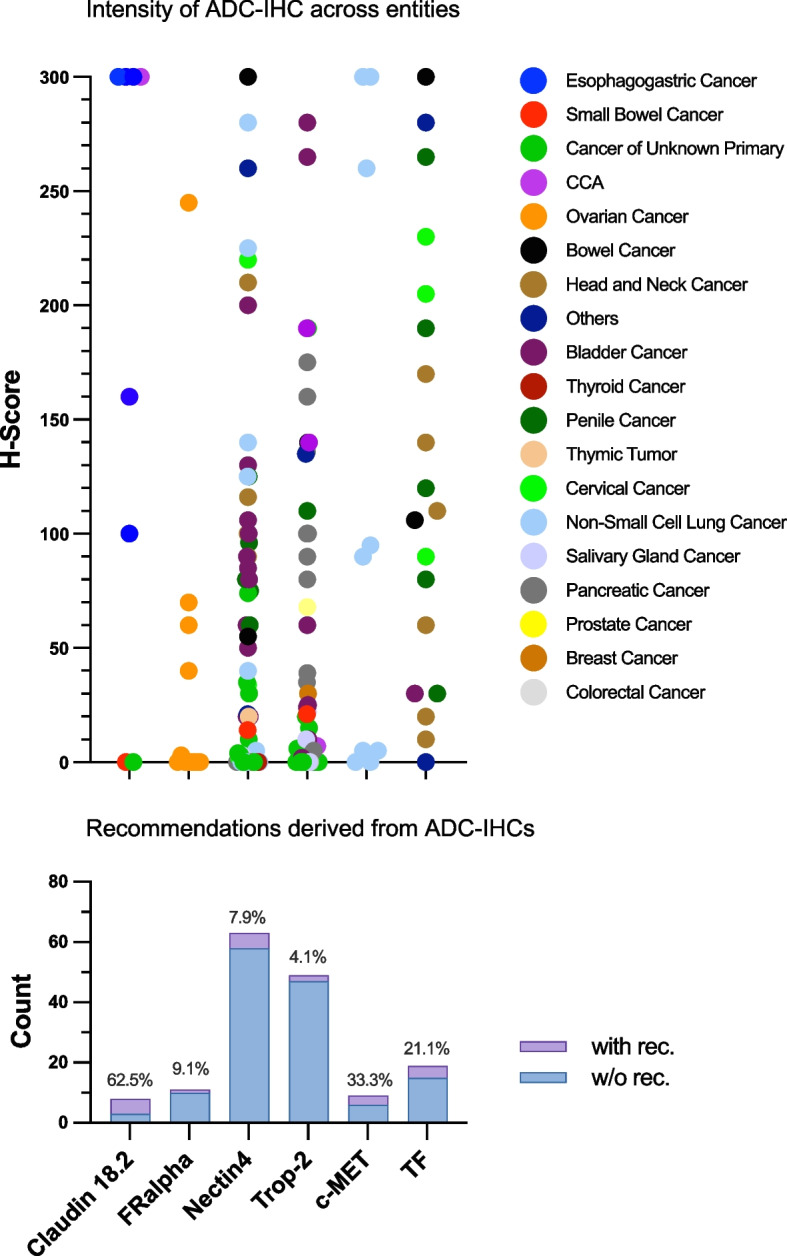
Supplementary diagnostics: ADC-IHC. The graph shows the number of ADC IHC assessments conducted for individual targets. The proportion of recommendations based on each marker is highlighted in purple. In the upper part, the corresponding H-scores (ranging from 0 to 300) are plotted. The color of the data points represents different tumor entities

The evaluation of ADC-IHC testing revealed significant differences in therapy recommendation rates across biomarkers. Claudin-18.2-targeted therapy (Zolbetuximab) had the highest recommendation rate (5/8 tested cancers, 62.5%), followed by c-Met-targeted therapy (Telisotuzumab-vedotin) (3/9 tested cancers, 33.3%), TF-directed therapy (Tisotumab-vedotin) (4/19 tested cancers, 21.1%), FRα-based therapy (Mirvetuximab-soravtansine) (1/11 tested cancers, 9.1%), Nectin-4-directed therapy (Enfortumab-vedotin) (5/63 tested cancers, 7.9%), and Trop-2-based therapy (Sacituzumab-govitecan) (2/49 tested cancers, 4.1%). Claudin-18.2-directed therapy was predominantly recommended in esophagogastric cancer (4/5, 80%), while TF-based therapy was primarily considered for cervical cancer (2/4, 50% of recommendations). FRα testing was limited to ovarian cancer by preset testing criteria. Nectin-4- and Trop-2-targeted therapies were recommended across different tumor types, but without a clear trend. c-Met-directed therapy, tested only in NSCLC, showed a moderate recommendation rate (33.3%).

Higher H-scores were typically associated with ADC therapy recommendations, particularly for Claudin-18.2, where 4/5 cases with recommendations had an H-score of 300, and one had 160. In contrast, one tumor with low expression (H-score 100) was not given a recommendation. FRα-targeted therapy was only recommended for a single case of ovarian cancer with an H-score of 245, while lower-expressing tumors (H-score 0–70) did not receive recommendations for Mirvetuximab-soravtansine. Nectin-4- and Trop-2-based therapies followed similar trends, with recommendations at H-scores ≥ 190. c-Met-targeted therapy was consistently recommended for NSCLC with H-scores of 260–300. TF-based therapy was recommended for tumors with H-scores 205–300, though one tumor with H-score 280 did not receive recommendations.

### *Evaluation of the metagenomic parameter HRD (*Fig. 4*)*

**Fig. 4:**
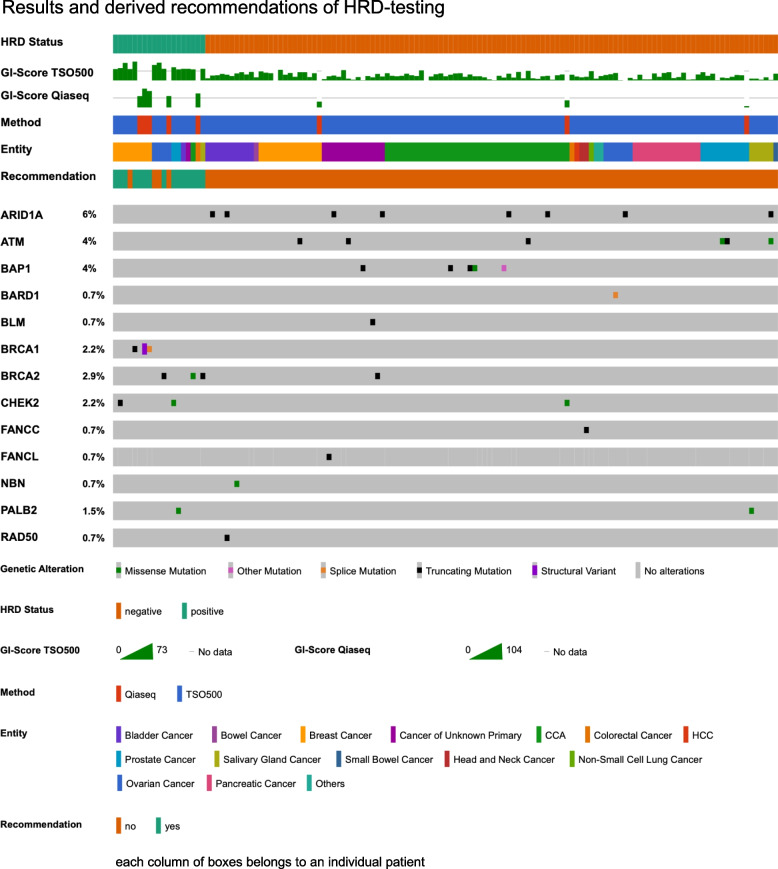
Supplementary diagnostics: HRD. This Oncoprint visualization presents all conducted HRD assessments, ranked according to their HRD status. HRD-positive cases are displayed on the left, while HRD-negative cases are positioned on the right. Two distinct testing methods were applied: TSO 500 (blue) and QIA (red). Additionally, the tumor entity and the resulting molecular tumor board (MTB) recommendations are depicted. The lower section illustrates the corresponding genetic alterations in key homologous recombination repair (HRR) genes, highlighting relevant genomic variations associated with HRD status

HRD testing revealed significant variability across tumor types. Using the TSO500-HRD (GIS ≥ 42) and Qiaseq Targeted DNA IO (GIS ≥ 56) panels, HRD positivity was highest in CRC at 50% (1 of 2 tested, limited interpretability), followed by ovarian cancer at 40% (4 of 10 tested) and breast cancer at 38% (8 of 21 tested). Prostate cancer and salivary gland cancer both exhibited HRD positivity rates of 16.7% (2 of 12 prostate cancers were positive, and 1 of 6 salivary gland cancers was positive).

Among frequently tested entities, CCA (*n* = 39) and pancreatic cancer (*n* = 14) showed low (1/39 CCA, 2.6%) to none (0/14 pancreatic cancers) HRD-positive cases. Rarely tested cancers, such as head and neck (*n* = 2), small bowel (*n* = 1), bowl (*n* = 1), HCC (*n* = 1), and SCLC (*n* = 1), also showed no HRD positivity.

HRR gene mutations were sometimes associated with HRD, particularly *BRCA1/2* in ovarian and breast cancers. A case of ovarian cancer with a *BRCA2* truncating mutation (GIS 45, TSO500) and a case of breast cancer with a *BRCA1* fusion (GIS 104, Qiaseq) were HRD-positive. However, a case of CUP with a pathogenic *BRCA2* S1970* truncation mutation was HRD-negative (GIS 25, TSO500), which we hypothesized to be possibly due to monoallelic occurrence.

Another HRR gene, *PALB2*, exhibited variable HRD scores in two cases. One case (prostate cancer, GI score TSO500: 43) received a recommendation based on its HRD status, whereas the other (salivary gland cancer, GI score TSO500: 7) did not receive a recommendation taking into account its negative HRD-status.

Other HRR genes, including *ARID1A* (*n* = 8), *ATM* (*n* = 6), *BAP1* (*n* = 5), *BARD1* (*n* = 1), *BLM* (*n* = 1), *FANCC* (*n* = 1) *FANCL* (*n* = 1), *NBN* (*n* = 1), and *RAD50* (*n* = 1) showed consitently negative HRD association.

### *Assessment of MMR-IHC, MSI-PCR and TMB (*Fig. 5a + *b)*

**Fig. 5:**
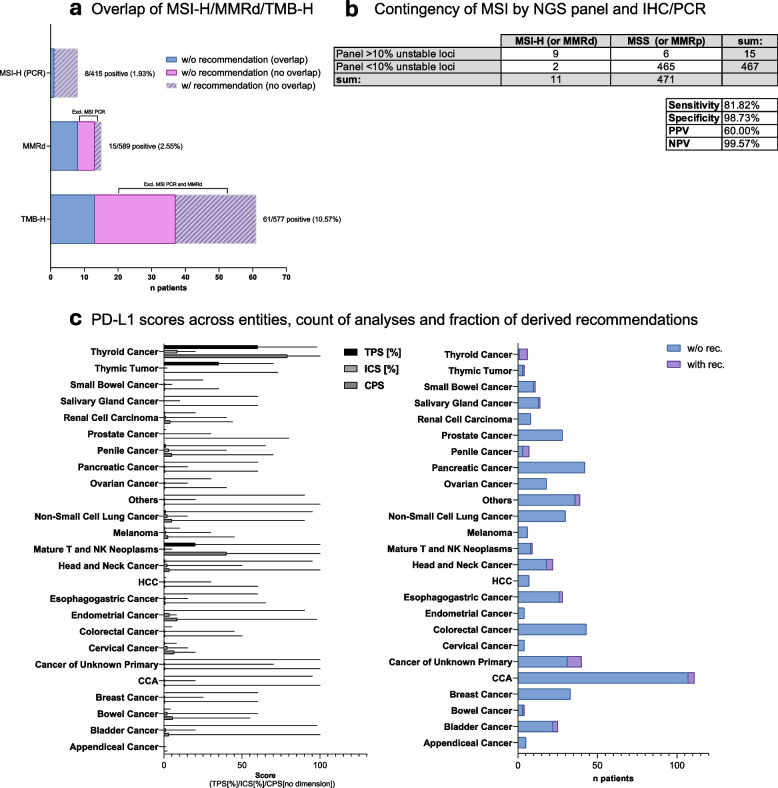
Supplementary diagnostics: MSI-High, MMRd, TMB-High, and PD-L1. a Illustrates the results of supplementary molecular diagnostics, including microsatellite instability high (MSI-High), mismatch repair deficiency (MMRd), and tumor mutational burden-high (TMB-H) assessments. The bar plot depicts the number of positive cases detected for each category: MSI-H, MMRd, and TMB-H, with the proportion of cases receiving molecular tumor board (MTB) recommendations indicated in hatched purple. The solid purple and hatched purple bars represent cases that were exclusively positive for a given parameter (e.g., MSI-H, MMRd, or TMB-H, i.e., no overlap or redundancy between testing methods was present) while hatched purple means a therapy recommendation was based on that specific non-overlapping and therefore non-redundant biomarker. b The contingency table illustrates MSI-H (or MMRd) and MSS (or MMRp) cases, assessed using two independent diagnostic methods (standard MSI PCR or MMR immunohistochemistry (MMR-IHC) and the percentage of unstable loci identified in panel-based diagnostics). Sensitivity, specificity, positive predictive value (PPV), and negative predictive value (NPV) are provided. c This figure presents PD-L1 expression levels across different tumor types, categorized by tumor proportion score (TPS, black), immune cell score (ICS, dark gray), and combined positive score (CPS, light gray), from top to bottom each. The left panel displays the distribution of PD-L1 scores for each tumor entity (median and maximum), while the right panel shows the number of cases assessed, stratified by the presence or absence of a MTB recommendation. The blue bars represent patients without therapy recommendation based on PD-L1 expression, whereas the purple segments indicate cases where PD-L1 positivity led to an MTB recommendation

MSI-PCR identified MSI-high (MSI-H) status in 8 of 415 cases (1.9%), with 87.5% (7/8) receiving ICI recommendations. MMRd IHC in 589 cases detected 15 MMR-deficient tumors (2.5%), including 8 not investigated by PCR due to unavailable normal tissue; 2 of these received ICI recommendations.

TMB-H (≥ 10 mutations/megabase) was found in 61 of 577 cases (10.6%), with 13 overlapping with MSI-H/MMRd and 48 in MSS/MMRp tumors. Among the latter, 24 received ICI recommendations. TMB varied by tumor type, with bladder cancer (9.7 (mean) ± 8,9 (SD)), melanoma (22.7 ± 46.9), and CRC (7.4 ± 10.1) showing TMB, while ovarian (3.3 ± 2.2), pancreatic (3.9 ± 2.5), and prostate cancers (3.0 ± 2.1) had lower values. Endometrial cancer displayed the highest variability (39.8 ± 58.8) (Additional file 1: Fig. S5).

In addition, we compared the different methods regarding their diagnostic overlap. MSI analysis by PCR and/or MMR protein IHC assessment were defined as the ground truth. Given this assumption, MSI-PCR remained the most specific biomarker for ICI selection, while MMR-testing identified additional cases, and TMB-H provided additional information in MSI/MMRd-negative tumors. Panel-based MSI assessment showed high specificity (98.7%) and NPV (99.6%) but had moderate PPV (60%) and 81.8% sensitivity, leading to false positive results. A tiered approach using panel diagnostics as a screening tool, with MSI-PCR confirmation for panel-positive cases, ensures accurate classification and prevents misclassification in MTB-guided therapy selection.

### *Examination of PD-L1 in the MTB context (*Fig. 5c*)*

The analysis of PD-L1 expression showed significant variability across cancer types, influencing ICI recommendations. Thyroid cancer (TPS = 58 (mean) ± 40.9 (SD), ICS = 8.9 ± 8.7, CPS = 68 ± 36.8) and penile cancer (TPS = 10.7 ± 24, ICS = 8 ± 14.2, CPS = 21.9 ± 28.2) had the highest PD-L1 scores, correlating with the highest ICI recommendation rates. Bladder cancer (TPS = 13.2 ± 30.2, ICS = 3.8 ± 5.6, CPS = 17.4 ± 31.3), CUP (TPS = 11.9 ± 26.3, ICS = 6.6 ± 15.7, CPS = 17.8 ± 31.9), Melanoma (TPS = 2.7 ± 4.1, ICS = 5.8 ± 11.9, CPS = 9.4 ± 17.5), and NSCLC (TPS = 20.7 ± 32.7, ICS = 4.1 ± 4.9, CPS = 15.4 ± 28.8) exhibited moderate-to-high PD-L1 expression, while breast cancer (TPS = 2.1 ± 10.4, ICS = 3.1 ± 5.7, CPS = 5.7 ± 12.1) and pancreatic cancer (TPS = 2.1 ± 9.7, ICS = 1.6 ± 3.3, CPS = 4.9 ± 12.3) had low levels.

ICI recommendations were highest in thyroid cancer (83.3%) and penile cancer (57.1%), moderate in CUP (22.5%) and head and neck cancer (18.2%), but low in bladder cancer (12%) despite high PD-L1 expression, mostly due to prior ICI. Melanoma and NSCLC received no ICI recommendations despite elevated PD-L1 scores, primarily because it had already been used in prior treatment.

### *Implemented treatments (*Fig. 6*, *Table 2*, and *Additional file 1*: Fig. S6)*

**Fig. 6:**
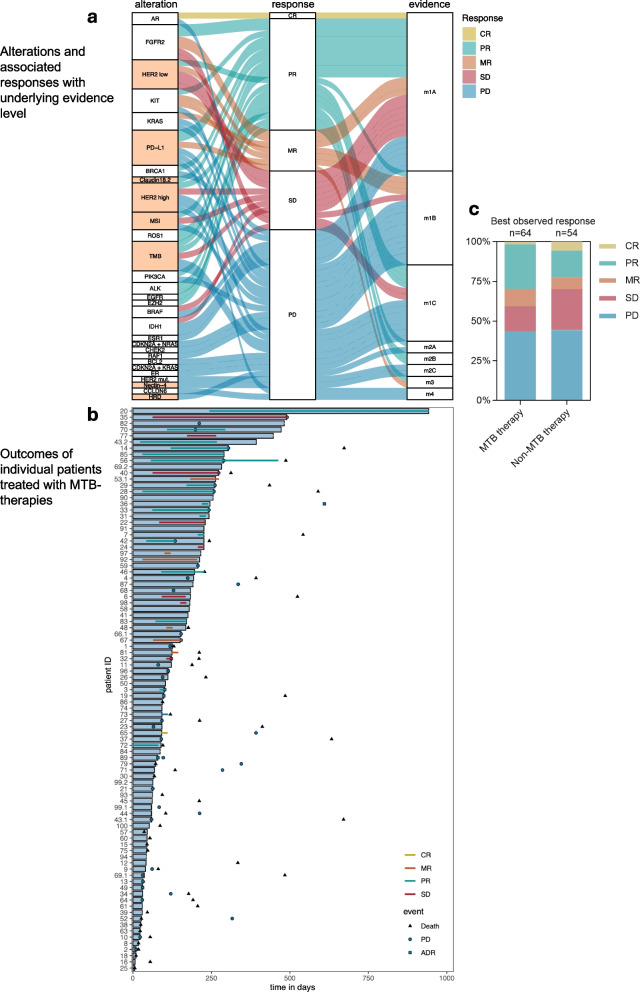
Employed treatments. a The alluvial plot on the left illustrates the biomarker used for therapy recommendation, with the clinically observed response displayed in the middle, and the corresponding evidence level on the right. b The swimmer plot on the right provides a detailed breakdown of individual therapy courses, illustrating treatment duration, response, and key clinical events. Patients with insufficient therapy data were excluded from this visualization. Each patient is assigned an anonymized ID. The white bars represent the duration of therapy, while the colored bars indicate the observed response: complete remission (CR) in yellow, mixed response (MR) in orange, partial response (PR) in blue, and stable disease (SD) in red. Additionally clinical events, including disease progression (PD), adverse drug reactions (ADR), and death are plotted. c Stacked bar charts of responses MTB therapy versus Non-MTB therapy

**Table 2:** Overview of patients receiving MTB-recommended therapies

Pat ID	Cancer type	Board recommendation	Rationale	EL	L (time of MTB/ EMA)	R	ADR	Sequential MTB therapy	PFS MTB Therapy	PFS prior therapy	PFS 2 / PFS 1 MTB
1	Melanoma	Trametinib	RAF1; BRAF	m1B	3	PD			118		
2	CCA	Binimetinib + Capecitabine	KRAS	m1C	0		Nausea		10	101	0.099
3	Melanoma	Nivolumab	**TMB-H**; POLE; POLD1	m1A	1	PR	Skin toxicity		101		
4	CCA	Ivosidenib	IDH1	m1A Z(FDA)	0	PD			174	89	1.955
5	Breast cancer	Pertuzumab + Trastuzumab	**HER2**	m1A	6						
6	Colorectal cancer	Sotorasib	KRAS	m1A	1	PD			168		
7	Esophagogastric cancer	Nivolumab	**PD-L1**	m1A	1	PR					
8	Mature T and NK neoplasms	Venetoclax + Ruxolitinib	BCL2	m1C	0	PD			17		
9	Esophagogastric cancer	Trastuzumab Deruxtecan	**HER2**	m1B	5	PD			61	181	0.337
10	Pancreatic cancer	Olaparib	BRCA1	m1A	4	PD			23		
11	Colorectal cancer	Sotorasib	KRAS	m1B	3	PD	Gastrointestinal side effects		80		
12	Salivary gland cancer	Pembrolizumab	**PD-L1**	m2A	0				44		
13	Melanoma	Binimetinib + Ribociclib	CDKN2A; NRAS	m1B	4	PD			32	468	0.068
14	Salivary gland cancer	Alpelisib + Androgen blockade	PIK3CA	m1C	4	PR			304		
15	Cancer of unknown primary	Olaparib	ATM	m2B	0				42		
16	Others	Azacitidine	TET2	m2B	1				7		
17	CCA	Pembrolizumab + Lenvatinib	**PD-L1**	m1B	5						
18	Esophagogastric cancer	FLOT + Trastuzumab	**HER2**	m2A	4				10		
19	Pancreatic cancer	Palbociclib + Trametinib	CDKN2A; KRAS	m1C	1		Gastrointestinal side effects (recurrent pancreatitis)		98		
20	Others	Tazemetostat	EZH2	m2A	6	PR			942	256	3.68
21	Colorectal cancer	Trastuzumab Deruxtecan	**HER2**	m1B	2	PD			63		
22	CCA	Ivosidenib	IDH1	m1A Z FDA	1	PD					
23	Cancer of unknown primary	Trastuzumab	**HER2**	m2C	0	PD			76		
24	Colorectal cancer	Pembrolizumab	**MSI-H**	m1A Z (EMA)	0	SD			82		
25	Breast cancer	Trastuzumab Deruxtecan	**HER2**	m1A	2				6	386	0.016
26	Others	Pembrolizumab	**PD-L1**	m1C	0		Dermal drug reaction		94		
27	CCA	Pembrolizumab + Lenvatinib	**PD-L1**	m1B	5	PD			92	471	0.195
28	Non-small cell lung cancer	Lorlatinib	ALK	m3	3	PR			258	699	0.369
29	Non-small cell lung cancer	Lorlatinib	ROS1	m1C, m3	2	PR			261		
30	CCA	Binimetinib + Capecitabine	KRAS	m1C	1				66		
31	Endometrial cancer	Pembrolizumab	**MSI-H; TMB-H**	m1A Z	0	PR			366		
32	CCA	Erdafitinib	FGFR2	m1B	0	SD	Onycholysis		122		
33	Head and neck cancer	Pembrolizumab	**PD-L1**	m1A (Z EMA)	0	PR			242		
34	Non-small cell lung cancer	Atezolizumab + Bevacizumab + Carboplatin	**TMB-H**	m1C	0	PD			120	184	0.652
35	Breast cancer	Trastuzumab Deruxtecan	**HER2**	m1A Z (FDA)	6	SD	Pneumonitis		491		
36	Non-small cell lung cancer	Atezolizumab + Carboplatin + Etoposide	**TMB-H**	m2B (Z FDA)	0	PR	Bullous pemphigoid (CTC Grade IV)		363		
37	CCA	Pembrolizumab	**MSI-H; TMB-H**	m1B Z	1	PD			90		
38	Mature T and NK neoplasms	Ruxolitinib	JAK3; STAT5B	m1B	0				25	304	0.082
39	Colorectal cancer	Inavolisib (CRAFT trial)	PIK3CA	N/A	3				30		
40	CCA	Futibatinib	FGFR2	m1C	1	SD			273		
41	Mature T and NK neoplasms	Ruxolitinib	STAT5B	m2B	0				175		
42	CCA	Pemigatinib	FGFR2	m1A Z	0	PR			134		
43	Others	Regorafenib	KIT	m1B Z	2	PD		Riptretinib	59		
44	Endometrial cancer	Pembrolizumab	**MMRd**	m1A Z	0				59	206	0.286
45	Colorectal cancer	Atezolizumab	**TMB-H**	m1C	5	PD			92		
46	Non-small cell lung cancer	Trastuzumab Deruxtecan	**HER2**	m1B	4	PR	Nausea		196	196	1
47	Cancer of unknown primary	Pembrolizumab + Carboplatin + Paclitaxel	**PD-L1**	m2A	0				244		
48	Esophagogastric cancer	Trastuzumab Deruxtecan	**HER2**	m1B	7	MR			168		
49	Others	Pembrolizumab	**TMB-H**	m2A Z	0		GI and dermatological adverse events		31	90	0.344
50	Pancreatic cancer	Pembrolizumab	**MSI-H; TMB-H**	m2A Z (FDA)	1		Nausea, vomiting		124	121	1.025
51	Non-small cell lung cancer	Lorlatinib	ALK	m3	4						
52	Thyroid cancer	Pembrolizumab + Lenvatinib	**PD-L1**	m1B	0					62	0
53	Melanoma	Imatinib	KIT	m1B	2	MR		Palbociclib	274		
54	Cancer of unknown primary	Capecitabine + Binimetinib	NRAS	m1B	0				179		
55	Prostate cancer	Niraparib + Abiraterone	CHEK2	m1B	3	PD				335	0
56	Cancer of unknown primary	Trastuzumab Deruxtecan	**HER2**	m2B Z is(IHC)	1	PR			463		
57	Breast cancer	Trastuzumab + Pertuzumab + Paclitaxel	**HER2**	N/A	6				46		
58	CCA	Pemigatinib	FGFR2–AHCYL1 fusion	m1A Z	1				179		
59	Colorectal cancer	Atezolizumab + Pertuzumab + Trastuzumab	**HER2**	m1A	2				105		
60	Breast cancer	Trastuzumab Deruxtecan	**HER2**	m1B	4				45		
61	Ovarian cancer	Everolimus + Bevacizumab	PIK3CA	N/A	5	PD			30	652	0.046
62	CCA	Olaparib	BAP1	m1C						181	0
63	Non-small cell lung cancer	Lorlatinib	EZR–ROS1 fusion	m1A	3				23		
64	Pancreatic cancer	Trastuzumab Deruxtecan	**HER2**	m1C/m2B	6	PD			30	96	0.313
65	Others	Androgen deprivation therapy (ADT)	AR	m1A		CR	Hyperglycemic decompensation, edema, hot flashes, pruritus, depressive mood, reduced strength		91	87	1.046
66	Bladder cancer	Brimigimadlin (Brightline trial, LMU)	MDM2 CNV	N/A	2	PD		Everolismus + Pazopanib	153		
67	CCA	Futibatinib + Pemigatinib	COL25A1–FGFR2 fusion	m1A Z	2	MR			150		
68	Ovarian cancer	Trastuzumab Deruxtecan	**HER2**	m2A Z is	6				183		
69	Breast cancer	Elacestrant	ESR1	m1A Z	5	PD		Trastuzumab Deruxtecan	51		
70	Non-small cell lung cancer	Lorlatinib	CD74–ROS1 fusion	m1A	1	PR			294	91	3.231
71	Thyroid cancer	Dabrafenib	BRAF	m1A	3	PD			68		
72	Breast cancer	Olaparib	BRCA1	m1A		PR			81		
73	Pancreatic cancer	Palbociclib + Trametinib	CDKN2A; KRAS	m1C	2	PD			92		
74	Ovarian cancer	BNT21-01 trial	CLDN6	N/A	4	PD			92	139	0.662
75	Cancer of unknown primary	Olaparib	**HRD**	m2A Z	1	PD			407		
76	Bowel cancer	Enfortumab Vedotin	**NECTIN4**	m2C	3				189	141	1.34
77	CCA	Ivosidenib	IDH1	m1A Z	2	SD			447		
78	Salivary gland cancer	Trastuzumab Deruxtecan	**HER2**	m1C Z	1						
79	Others	Pembrolizumab	**PD-L1**	m1A	3				72		
80	Breast cancer	Talazoparib	BRCA1; **HRD**	m1A	1						
81	Others	Ponatinib	KIT	m3	5	MR			124		
82	Melanoma	Immune checkpoint inhibitor	**TMB-H**	m1A Z		SD			482		
83	Cancer of unknown primary	Alectinib	LASP–ALK fusion	m1C-m2A	2	PR			171		
84	Colorectal cancer	Trastuzumab + Pertuzumab	**HER2**	m1A	4	SD			86		
85	Non-small cell lung cancer	Afatinib	EGFR	m1C		PR					
86	Cancer of unknown primary	Erlotinib	EGFR	m2B					23		
87	Others	Anastrozole	ER	m1C is	1	PD			191		
88	Non-small cell lung cancer	Immune checkpoint inhibitor	**PD-L1**	m1C	1				0		
89	Thyroid cancer	Pembrolizumab + Lenvatinib	**PD-L1**	m1B	1	MR	Colitis, suspected nephritis		79		
90	CCA	Futibatinib	FGFR2	m1A Z	2	PR			255		
91	Breast cancer	Trastuzumab Deruxtecan	**HER2**	m1A Z is	4	SD			257		
92	CCA	Futibatinib	FGFR2	m1A Z	1	MR			204		
93	Penile cancer	Immune checkpoint inhibitor	**PD-L1**	N/A	1	PD			69	60	1.15
94	Breast cancer	Trastuzumab Deruxtecan	**HER2**	m1A Z EMA	12	SD			43		
95	Cancer of unknown primary	Pembrolizumab	**MMR-d; TMB-H**	m2A Z			Autoimmune nephritis				
96	Esophagogastric cancer	Zolbetuximab	**CLDN18.2**	m1A Z is	1	PR			112		
97	Colorectal cancer	Adagrasib + Cetuximab	KRAS	m1A	10	MR			216		
98	Others	Binimetinib followed by Cobimetinib	BRAF	m1C		SD			181		
99	Salivary gland cancer	Goserelin + Bicalutamide	AR	m1B		PD		Enfortumab-Vedotin	83		
100	Non-small cell lung cancer	Carboplatin + Pemetrexed	KRAS	m1B					417	763	0.547

*EL* evidence level (ZPM scheme), *R* response, *L* line of therapy, defined as the minimum deducible treatment line from available clinical records at the time of MTB discussion, *ADR* adverse drug reaction, *CCA* cholangiocarcinoma, *CUP* carcinoma of unknown primary, *CRC* colorectal cancer, *NSCLC* non-small cell lung cancer, *SD* stable disease, *PD* progressive disease, *PR* partial remission, *MR* mixed response, *MSI* microsatellite instability, *IHC* immunohistochemistry, *FISH* fluorescence in situ hybridization. Defined “supplementary diagnostics” biomarkers are written in bold

Finally, we describe the therapies implemented and the corresponding clinical outcomes.

Therapies were implemented based on heterogenous biomarkers, most frequently HER2, PD-L1, MSI/MMR, FGFR2, and TMB. In total 64 MTB-guided tretment courses were evaluable. Among these, 1 (1.6%) achieved a complete remission (CR; here it needs to be added that the patient received radiotherapy in addition to the molecularly targeted drug), 18 (28.1%) a partial remission (PR), 7 (10.9%) a mixed response (MR), 10 (15.6%) stable disease (SD), and 28 (43.8%) progressive disease (PD), resulting in a clinical benefit rate (CBR = CR + PR + SD) of 45.3% (29 out of 64 response evaluable cases). In comparison, patients who received non-MTB recommended therapies achieved a CBR of 48.2% (26 out of 54 response evaluable cases), with 3 complete responses (5.6%), 9 partial remissions (16.7%), 4 mixed reponses (7.4%), 14 cases of stable disease (25.9%), and 24 cases of progressive disease (44.4%). Of the MTB-implemented therapies, 44% were initiated based on supplementary biomarkers diagnostics, while the remaining 56% were derived from conventional panel sequencing results.

Most MTB-guided therpies were recommended at high levels of evidence (m1A, m1B) and were associated with higher response rates, although responses were also observed in cases with lower-evidence recommendations.

Median progression-free survival (PFS) was 8.4 months (257 days) in the MTB-guided cohort and 5.4 months in the patients treated with Non-MTB therapies (165 days). Median overall survival (OS) was 6.4 months in patients who either continued their existing therapy or received no additional therapy (“no therapy”-group), 13.6 months in patients who received MTB-guided therapies, and 15.6 months in patients treated with alternative, non-MTB-directed regimes. These findings represent descriptive associations within the cohort and should not be interpreted as causal effects.

Adverse drug reactions (ADR) were documented in 14% of the implemented MTB therapies, most frequently in patients treated with immune checkpoint inhibitors (ICI) and antibody drug conjugates (ADC). ADR were recorded if reported in discharge letters or clinical documentation, irrespective of grade, and did not necessarily lead to treatment discontinuation.

## Discussion

The findings of this study emphasize the evolving role of supplementary diagnostic methodologies in precision oncology and their impact on MTB-guided therapy recommendations. Our results demonstrate that the integration of HRD testing, ADC-IHC, HER2 IHC and FISH, MSI assessment via PCR and IHC, and PD-L1 IHC contributed to a broader spectrum of actionable findings. While conventional next-generation sequencing (NGS) panel diagnostics remain the foundation of MTB decision-making, the addition of these diagnostic tools enhanced the overall rate of clinically relevant recommendations. In absolute numbers 37 implemented therapies were either fully or partially based on the testing and evaluation criteria defined as “supplementary” in this study, making up a significant proportion of 37% of all given therapies.

The clinical value of HRD testing was most evident in ovarian and breast cancer, where HRD positivity was observed at rates of 38.5% and 25.0%, respectively, consistent with literature [36]. However, HRD testing also identified potentially actionable cases in CUP and bladder cancer, leading to off-label treatment recommendations. The scarcity of HRD-positive cases in CCA aligns with the existing literature [36], reinforcing the limited relevance of HRD testing in this entity. Importantly, discrepancies between HRD positivity and HRR gene mutations, such as *BRCA1/2* and *PALB2*, suggest that these genomic alterations alone may not entirely reflect HRD status, highlighting the need for functional HRD assessments in these cases besides ovarian and breast cancer. This discrepancy underscores that not all HRR gene mutations lead to HRD and that further investigation is required to determine their exact relevance. Notably some mutations, though detected by NGS, may not have functional impact despite being detected in sequencing assays, leaving their pathogenicity uncertain. Additionally, HRD status may not be affected if these mutations are present in a monoallelic state rather than biallelic inactivation, which is often necessary to completely abolish HRR.

It should also be considered that our study was based on panel diagnostics rather than whole-genome or whole-exome sequencing, where HRD assessment is not routinely included. Currently, from economic, practical, and technical perspectives, panel diagnostics remain the more feasible approach [37], as also applied by MTBs. Consequently, HRD analysis is gaining furher relevance within this framework, at least until more advanced methods become widely adopted.

The incorporation of ADC-IHC across various cancer entities further enhanced the spectrum of discussed targets. Claudin-18.2 testing yielded the highest recommendation rate (62.5%), predominantly in esophagogastric cancer. Similarly, Nectin-4 and TF showed selective utility in bladder, salivary gland, cervical, and head and neck cancers, supporting the rationale for ADC biomarker testing in these entities. However, the Trop-2 and FRα biomarkers provided comparatively low recommendation rates. It should be noted that for some ADC-targets the predictive value of IHC has yet to be fully determined. For example, in case of Sacituzumab govitecan in the first-in-human trial in various solid tumors Trop-2 expression was only available in few cases, where partial remissions were observed in tumors with weak, moderate, and high Trop-2 expression, while a stable disease was also documented in a tumor negative for Trop-2 IHC [38]. In addition, in the indication of triple-negative breast cancer, for which Sacituzumab govitecan was approved, most archival tumor specimens tested in a clinical trial were moderately to strongly positive [39], hence precluding a definitive evaluation of the biomarkers. For this purpose, the recommendations for Trop-2 targeted therapies in our MTB were cautiously based on a biological rationale only (m4). The same restriction applied to Enfortumab vedotin, whose approval for urothelial cancer was also not biomarker dependent, since the majority of tumors exhibited a strong staining of Nectin-4 [32]. This demonstrates the need of an ongoing evaluation of the corresponding biomarkers which is mirrored by low recommendation rates in our study. In contrast, Telisotuzumab-vedotin, Mirvetuximab-soravtansine, and Zolbetuximab with a proven record of strong biomarker dependency [26, 40, 41] were employed consistently in the respective entities and resulted in higher evidence levels. Therefore, especially c-Met, FRα, and Claudin18.2 should be given priority in NSCLC, ovarian cancer, and esophagogastric cancer, respectively, in an MTB if not already tested previously.

The next addition to panel diagnostics we focused on was MSI. Our comparative analysis of MSI detection methods underscores the complementary nature of MSI-PCR, MMR IHC, and sequencing-based MSI inference. While MSI-PCR remains the gold standard, primarily in CRC, we demonstrate that MMR IHC identifies additional cases that may not be captured by PCR-based methods, particularly in situations where normal tissue comparison is unavailable. Conversely, panel-based MSI detection (cut-off 10% unstable loci) exhibited high specificity (98.7%) and a strong negative predictive value (99.6%), making it a useful tool for ruling out MSI-high cases. However, its moderate positive predictive value (60%) indicates that confirmatory testing is essential for panel-positive cases to avoid false-positive MSI classifications. Moreover, the panel-based method also missed positive cases in view of its sensitivity of 80%. These findings support a tiered MSI testing approach, wherein panel diagnostics serve as a screening tool, with MSI-PCR and MMR IHC providing confirmatory validation in cases of uncertainty.

TMB analysis revealed significant variability across tumor entities, with high TMB values observed in bladder cancer, melanoma, and MSI-H CRC, consistent with their established immunogenic profiles [42]. However, the clinical utility of TMB as an independent biomarker remains complex, as evidenced by the fact that half of the TMB-high cases (≥ 10 mut/Mb) in our cohort did not receive ICI recommendations. This discrepancy can be explained by additional clinical considerations, such as tumor histology, prior treatment history, and the presence of co-occurring MSI influencing ICI eligibility. Among those patients who received therapy, three had documented clinical benefit (2 PR and 1 SD), while two progressed. Despite the entity-agnostic approval by the Food and Drug Administration in the USA based on the KEYNOTE-158 trial [43], the exact cut-off value remains an area of investigation and some tumor types may benefit less, necessitating an ongoing investigation of achievable real-world outcomes [44]. Altogether, an evaluation of TMB should be incorporated into MTB discussions to enhance access to ICI for select patients.

The same also applies to PD-L1-IHC. Notably, thyroid and penile cancers as well as CUP and head and neck squamous cell carcinomas achieved particularly high ICI recommendation rates based on this biomarker. Less frequently, recommendations were also given to cases of CCA, bladder cancer, and esophagogastric cancer ( the latter two often with previous ICI). However, only a few therapies were implemented with available follow-up.

The evaluation of HER2 alterations highlights the expanding role of HER2-low status in treatment selection. While both HER2-low and amplified cases were primarily recommended for trastuzumab- and ADC-based therapies, HER2-low findings constituted the majority of recommendations in cancers such as CCA, breast, and prostate cancer. This shift reflects the emerging recognition of HER2-low as a distinct therapeutic category even in tumors beyond breast and gastric cancer, and this is also the subject of current research (NCT04482309). The comparison of *HER2 (ERBB2)* copy high specificity (99.2%) but moderate sensitivity (60.0%), underlining the necessity of at least additional IHC in every case because 40% of *HER2* amplified cases are missed by current NGS testing. The most likely reason for the low sensitivity is that sequencing-based CNV assessment can be affected by tumor heterogeneity and low tumor purity. The addition of IHC and FISH testing provides critical sensitivity enhancements to ensure accurate HER2 classification. Moreover, the definition of a HER2 low category has made HER2 IHC indispensable, since it cannot be inferred from panel sequencing and FISH analysis.

From a therapeutic standpoint, our analysis of MTB-guided therapies stresses the value of integrating high-evidence biomarker-driven treatments, while remaining open to lower-evidence strategies in select cases. Therapies based on m1A and m1B evidence levels dominated the recommendations, and high-evidence-level recommendations tended to be associated with favorable clinical outcomes.

Although the findings are promising, the study has significant limitations. First, the real-world nature of MTB decision-making introduces variability in treatment implementation, influenced by factors such as patient comorbidities, prior treatment failures, and access to off-label therapies. Additionally, the retrospective nature of some analyses may limit causal inferences between biomarker findings and therapeutic outcomes. Future studies should incorporate prospective validation of supplementary diagnostics and evaluate their impact on long-term survival outcomes. Furthermore, while this study analyzed the incremental utility of supplementary diagnostics, it remains unclear whether their widespread adoption would significantly alter standard-of-care approaches in a cost-effective manner. The financial and logistical feasibility of integrating these biomarkers into routine MTB workflows warrants further exploration.

Given the high rate of implemented therapies based on supplementary diagnostics, it is worthwhile to compare other potential extensions of MTBs, such as WGS. While WGS enables comprehensive genomic analysis, its additional clinical utility compared to more targeted and cost-efficient methods remains uncertain. Many therapeutically relevant mutations are already covered by panel sequencing, and studies suggest that WGS rarely leads to changes in clinical management. Higher costs, longer turnaround times, and the associated resource demands may further limit its widespread implementation, although WGS could be beneficial in specific cases, such as the detection of complex structural rearrangements [45–47].

Based on our findings, we propose a pragmatic workflow for integrating supplementary diagnostics into MTB practice. HER2 and PD-L1 IHC should be routinely performed, as they are inexpensive, feasible on limited tissue, and can identify therapeutic opportunities not captured by NGS alone (e.g., HER2-low). The feasibility in this study is also evidenced by the number of samples that could be successfully analyzed using the defined supplementary methods in a real-world setting. HER2 amplification inferred from copy number gains should be confirmed by IHC and, if needed, FISH. TMB should be routinely assessed as part of NGS analysis, as it provides actionable information independent of MSI. MSI testing is best performed through a combination of MMR IHC and NGS, if necessary with PCR confirmation in positive cases to ensure accuracy. HRD testing should be restricted to entities where clinical relevance is established (e.g., breast, ovarian, pancreatic cancer), given that BRCA mutations may occur without HRD. Finally, ADC biomarker testing by IHC should be applied selectively in tumor types with clinical evidence or approvals, including Claudin-18.2 in gastric cancer, FRα in ovarian cancer, C-MET and Nectin-4 in non-squamous NSCLC, Nectin-4 and tissue factor in head and neck SCC, Nectin-4 and TROP-2 in urothelial cancer, and Nectin-4 and TROP-2 in CUP.

In conclusion, our study demonstrates that supplementary diagnostics—including HRD testing, ADC-IHC, HER2 IHC and FISH, MSI assessment via PCR/IHC, and PD-L1 IHC—enhance the spectrum of actionable findings and therapeutic recommendations in precision oncology. These methods complement conventional NGS panel diagnostics and provide additional stratification tools for patient selection, particularly in rare and under-characterized tumor types. Incorporating these biomarkers into MTB workflows marks a step toward a more comprehensive precision oncology framework, where multiple diagnostic modalities converge to optimize individualized cancer treatment.

Beyond outcome assessment, this study also serves as an internal evaluation of newly implemented modifications, allowing for a structured review of our evolving diagnostic strategies. By integrating novel biomarkers and refining analytical approaches, we aim to further optimize our MTB framework to enhance the precision and clinical relevance of off-label therapy recommendations.

## Conclusions

The integration of additional biomarker assessments into MTB workflows enhances precision oncology by expanding the pool of patients eligible for targeted therapies. Supplementary diagnostics such as HRD testing, ADC-IHC, HER2 IHC/FISH (including the reporting of Her2 low status), MSI, and PD-L1 analysis increased actionable findings beyond standard panel sequencing. These mostly cost-effective methods enabled additional treatment recommendations. Their routine use supports a broader, more adaptable, and patient-centered approach to individualized cancer care. Thus, rather than focusing solely on more comprehensive sequencing technologies such as whole-genome or whole-exome sequencing, the strategic implementation of additional techniques—particularly predictive IHC—should be considered a core component of modern MTB practice.

## Supplementary Information

Additional file 1: Table S1 & S2; Fig. S1-S6. Table S1: Additional testing selection criteria; Table S2: Antibodies for IHC-IHC; Fig. S1_1 and S1_2: ADC-IHC evaluation by H Score; Fig. S2: Sample information; Fig. S3: Recommendations based on entity + evidence levels; Fig. S4: Recommendations based on genetic alterations; Fig. S5: TMB distribution by entity; Fig. S6: Survival analyses.

## Data Availability

The datasets generated and/or analysed during the current study contain pseudonymised clinical and molecular data derived from routine patient care and prospective follow-up within a molecular tumour board programme. These data include sensitive health-related information and rare disease constellations, for which re-identification cannot be fully excluded despite pseudonymisation. In accordance with the approved research ethics protocol (protocol code 20-1682-101) and the informed consent obtained from study participants, public deposition of the full datasets in an open-access repository is not permitted due to ethical, legal and data protection constraints (EU General Data Protection Regulation, Art. 9). In particular, participants did not consent to unrestricted public data sharing, and the ethics approval explicitly prohibits transfer of individual-level data to third parties. Controlled access to anonymised and aggregated datasets may be granted for bona fide academic research purposes upon reasonable request, subject to review by the study investigators and approval by the responsible ethics and data protection bodies at the University of Regensburg and the University Hospital Regensburg. Requests should be addressed to the corresponding author (contact details provided in the manuscript). Data access is conditional upon the conclusion of a data use agreement specifying the purpose of use, prohibiting downstream data sharing and commercial use, and ensuring compliance with applicable data protection regulations. Requests will be reviewed within a reasonable timeframe (2-3 weeks).

## Abbreviations

ADC: Antibody–drug conjugate
ADR: Adverse drug reaction
AR: Androgen receptor
BSC: Best supportive care
BZKF: Bavarian Center for Cancer Research
CCA: Cholangiocarcinoma
CNV: Copy number variation
CPS: Combined Positive Score
CR: Complete response
CRC: Colorectal cancer
CUP: Cancer of unknown primary
EL: Evidence level
EMA: European Medicines Agency
FDA: Food and Drug Administration
FISH: Fluorescence in situ hybridization
FRα: Folate Receptor Alpha
GIS: Genomic Instability Score
HER2: Human Epidermal Growth Factor Receptor 2
HRD: Homologous Recombination Deficiency
HRR: Homologous Recombination Repair
ICI: Immune Checkpoint Inhibitor
ICS: Immune cell score
IHC: Immunohistochemistry
MMR: Mismatch repair
MMRd: Mismatch repair deficient
MMRp: Mismatch repair proficient
MR: Mixed response
MSI: Microsatellite instability
MSS: Microsatellite stable
MTB: Molecular tumor board
NGS: Next-generation sequencing
NSCLC: Non-small cell lung cancer
OS: Overall survival
PCR: Polymerase chain reaction
PD: Progressive disease
PD-L1: Programmed Death-Ligand 1
PFS: Progression-free survival
PR: Partial response
PSMA: Prostate-specific membrane antigen
QIA: QIAseq Targeted DNA Custom Panel
SD: Stable disease
TMB: Tumor mutational burden
TPS: Tumor proportion score
ZPM: Zentrum für Personalisierte Medizin
