# Supplementary material for: Supplementary biomarker testing in molecular tumor boards increases actionable therapy recommendations: a prospective real-world study of 658 patients
Source: BMC Med. 2026 Jan 14;24:50. doi: 10.1186/s12916-026-04636-y (PMC12849312; doi:10.1186/s12916-026-04636-y)
Supplement: Supplementary file 1 — Additional file 1: Table S1 & S2; Fig. S1-S6. Table S1: Additional testing selection criteria; Table S2: Antibodies for IHC-IHC; Fig. S1_1 and S1_2: ADC-IHC evaluation by H Score; Fig. S2: Sample information; Fig. S3: Recommendations based on entity + evidence levels; Fig. S4: Recommendations based on genetic alterations; Fig. S5: TMB distribution by entity; Fig. S6: Survival analyses. [file 12916_2026_4636_MOESM1_ESM.pdf]

Table S1: Additional testing selection criteria

Entity	Additional testing	Omitted testing
CUP	HRD, Nectin-4, Trop-2	-
Lung carcinoma	c-MET, Nectin-4	-
Breast carcinoma	HRD	-
Salivary gland carcinoma	HRD, Trop-2, Nectin-4, AR, PSMA	-
Ovarian carcinoma	HRD, Folate receptor α	-
Endometrial carcinoma	HRD, Folate receptor α, Trop-2	-
Pancreatic carcinoma	HRD, Trop-2	PD-L1
Prostate carcinoma	HRD	PD-L1
Urothelial carcinoma	HRD, Nectin-4, Trop-2	-
Lymphoma	-	MMR enzymes
Head and neck tumors (SCC)	Nectin-4, Tissue Factor	-
CRC	-	PD-L1
Gastric carcinoma	Claudin18.2	
CCA	HRD	-
Malignant melanoma	HRD	-

AR, Androgen receptor immunohistochemistry; HRD, homologous DNA repair deficiency analysis; CUP, Cancer of unknown primary; SCC, squamous cell carcinoma; CCA, cholangiocarcinoma; CRC, colorectal cancer; MMR, Mismatch repair enzymes, PD-L1, Programmed death-ligand 1.

Table S2: Antibodies for ADC-IHC

Antigen [clone if monoclonal antibody]	Dilution	Company	Catalog
Folate receptor α [FOLR1-2.1]	Ready to use	Ventana Medical Systems, Inc.	740-5065
Folate receptor α [BN3.2]	1:400	Novocastra	NCL-L-Fralpha
Claudin 18	1/200	LifeSpan BioSciences	LS-B16145
Tissue Factor [EPR22548-240]	1/1000	Abcam plc.	ab252918
c-Met [SP44]	1/200	Abcam plc.	ab227637
TROP-2 [SP294]	1/400	Abcam plc.	ab227690
Nectin-4 [EPR15613-68]	1/100	Abcam plc.	ab192033

Fig. S1_1

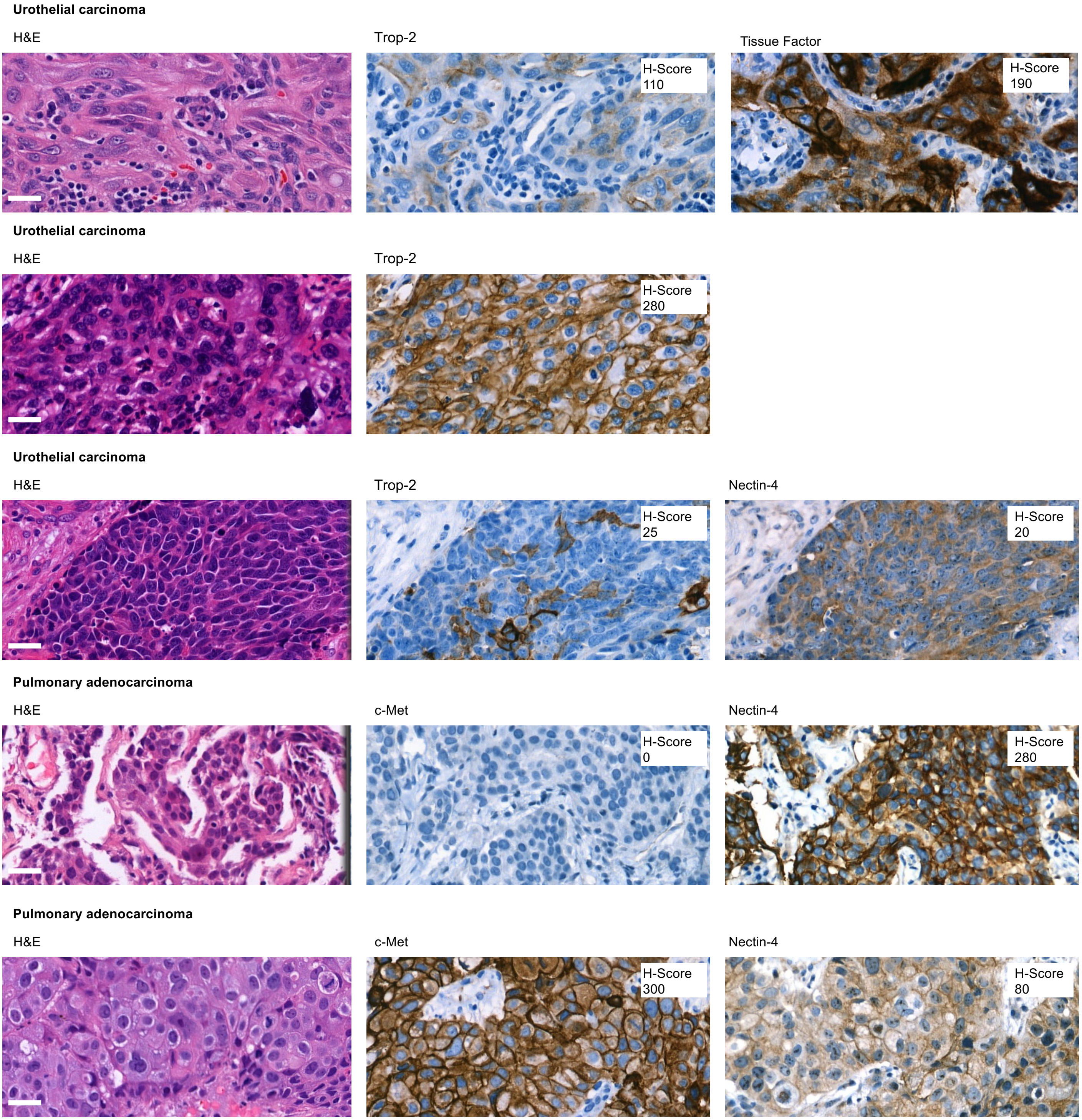

The figure continues on the next page

Fig. S1_2

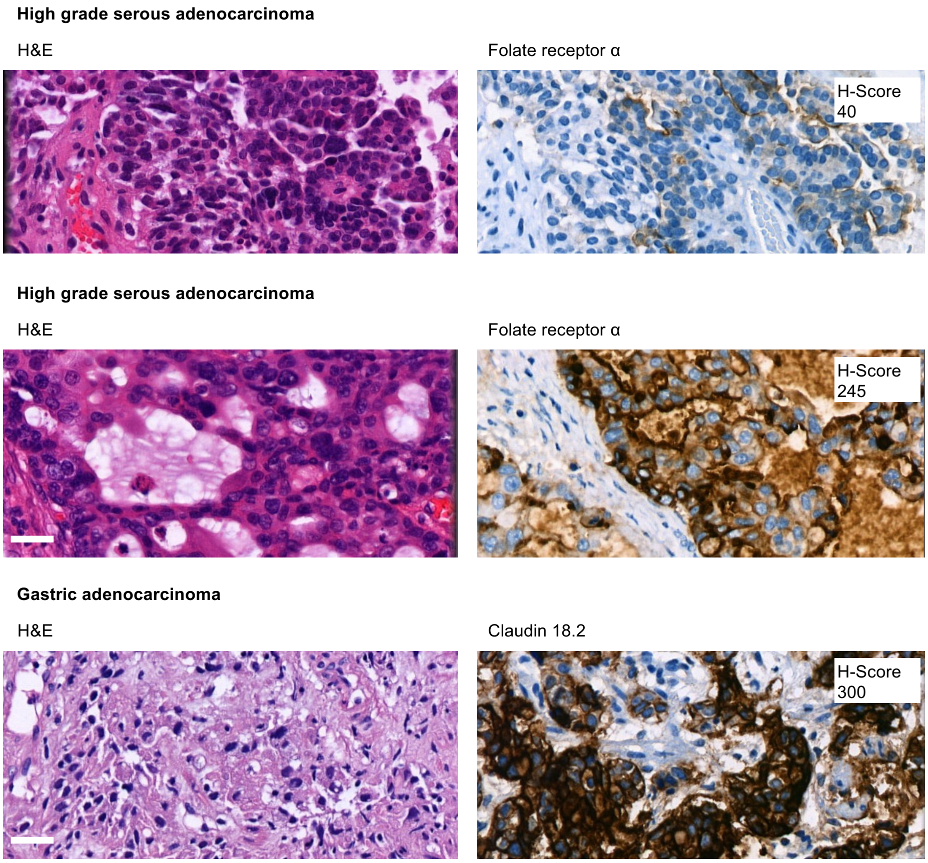

Fig. S1. ADC-IHC evaluation by H Score

Representative histological images showing H&E staining, ADC marker expression, and corresponding H-Score analysis in various tumor samples. The figure illustrates different staining intensities across urothelial carcinoma, pulmonary adenocarcinoma, high grade serous adenocarcinoma and gastric adenocarcinoma.

Fig. S2

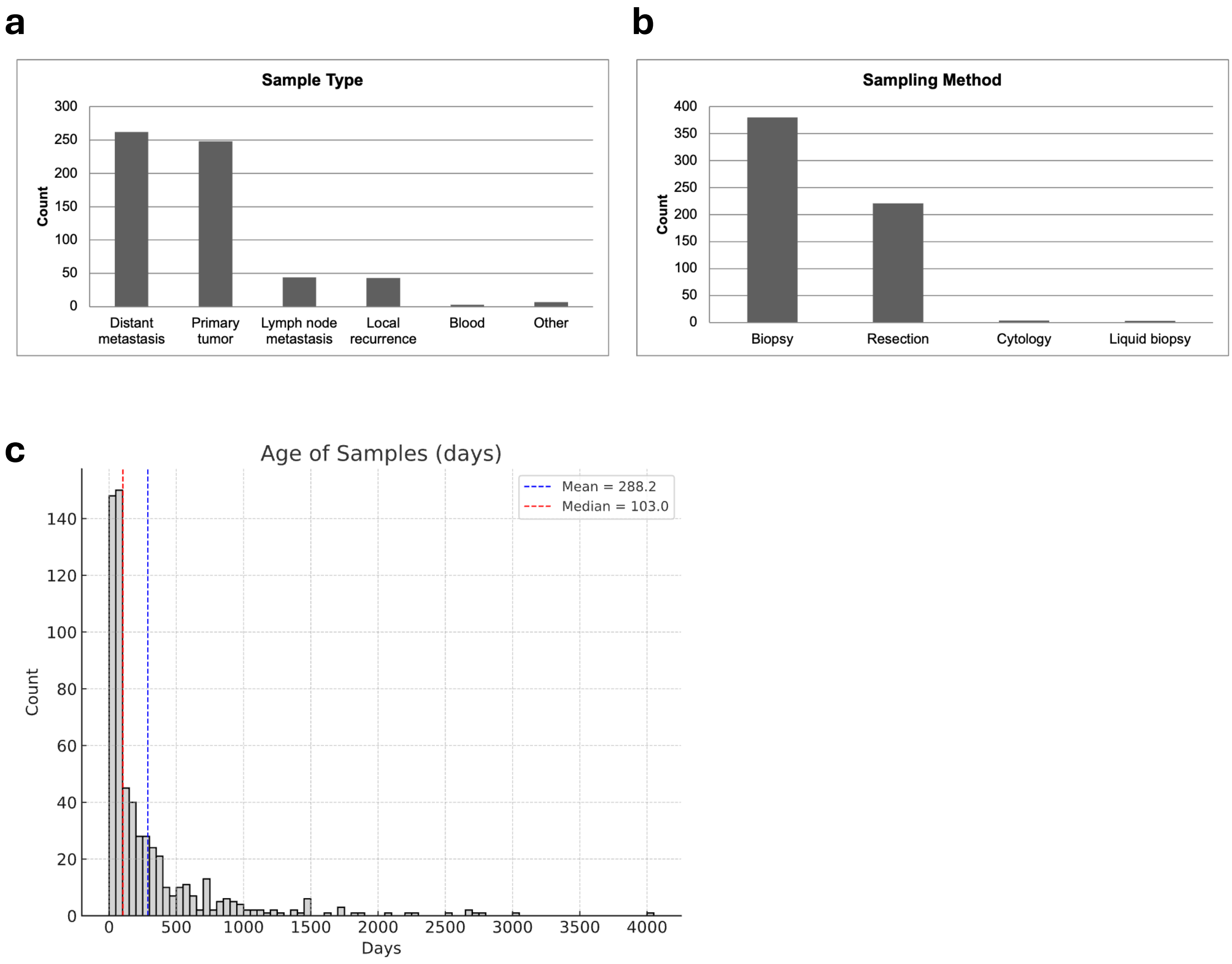

Fig. S2. Sample information
(a) Sample type as bar chart.
(b) Sampling method as bar chart.
(c) Sample age in days presented in intervals of 50 days with provided mean and median values.

Fig. S3

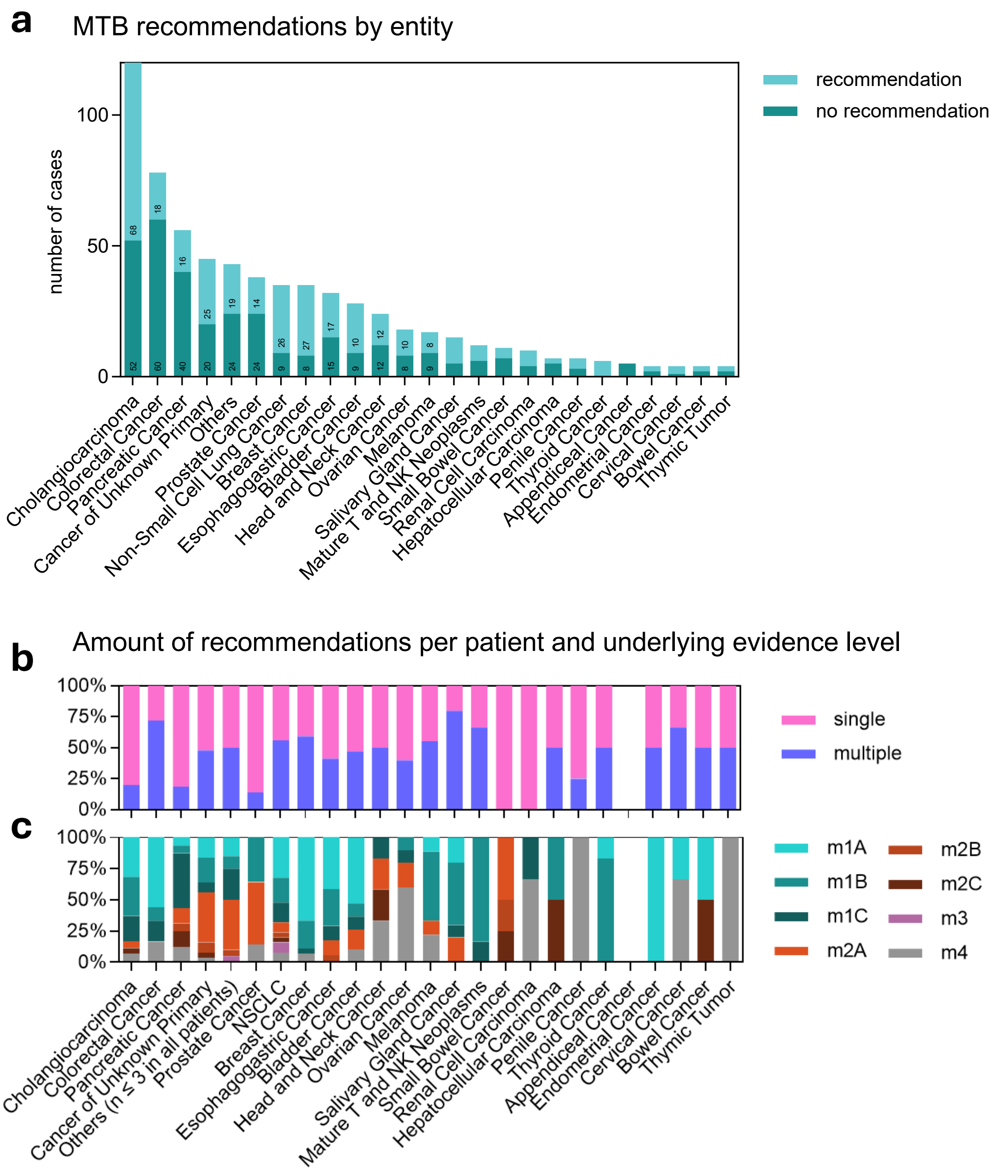

Fig. S3. Recommendations based on entity + evidence levels

(a) The bar plot illustrates the number of cases per tumor type, distinguishing between patients who received a molecular tumor board (MTB) recommendation (light green) and those who did not (dark green).

(b) The stacked bar plot categorizes cases in their respective tumor types based on whether a single (pink) or multiple (blue) recommendations were provided.

(c) The distribution of evidence levels (m1A–m4) for each tumor type is displayed. Study recommendations were classified as biological rationale (m4).

Fig. S4

Alterations by gene underlying MTB recommendations across entities

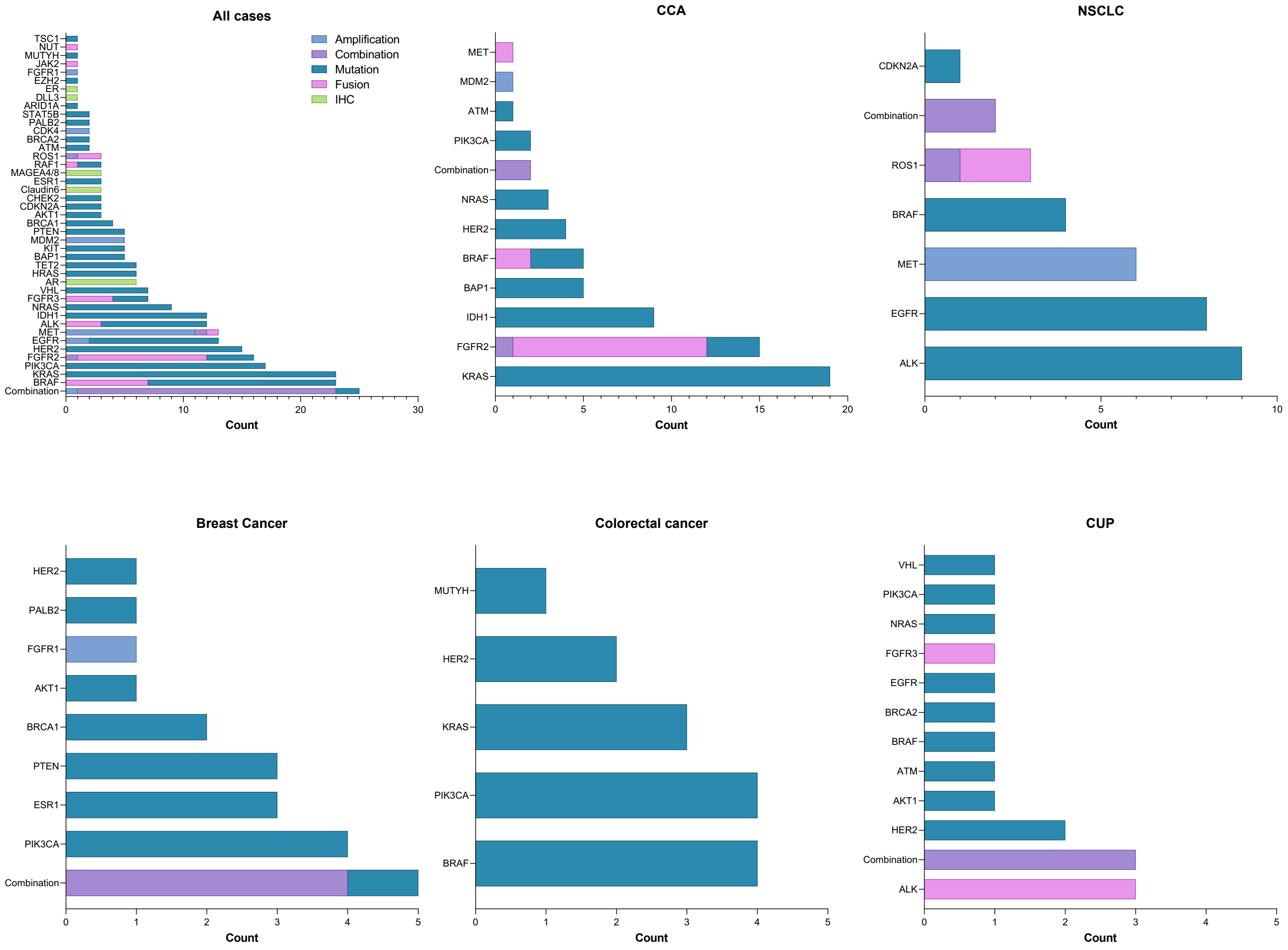

Fig. S4. Recommendations based on genetic alterations

The top-left graph provides an overview of the most common genetic alterations that led to a therapy recommendation. The different bar colors represent the type of alteration, with amplifications in light blue, mutations in blue, fusions in pink, IHC-based markers in green, and combinations marked in purple. The five additional graphs display the five most frequent tumor entities and their respective genetic alterations that resulted in therapy recommendations. HER2 IHC is not included in this graph, since it is shown more in detail in the main figures.

Fig. S5

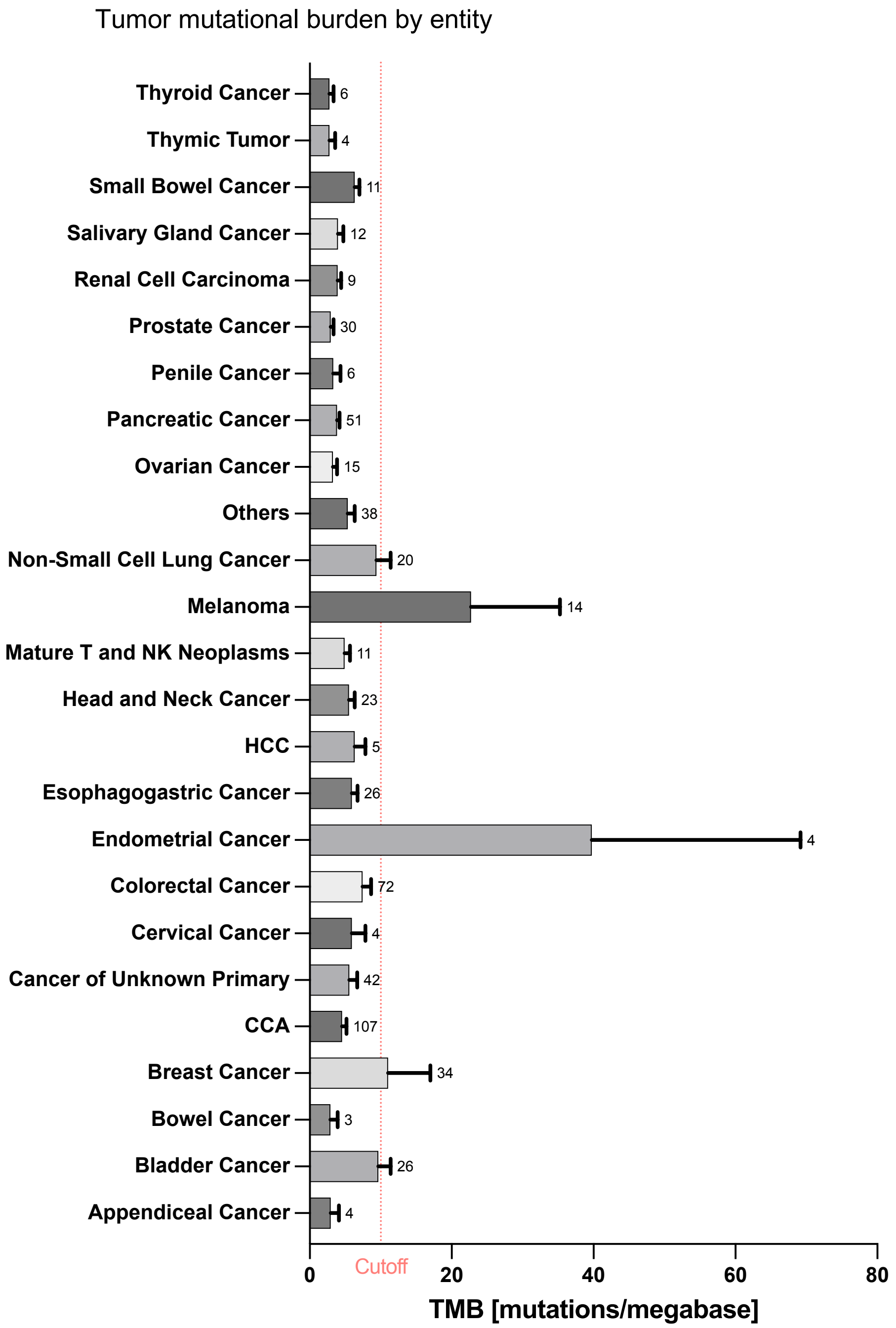

Fig. S5. TMB distribution by entity
The bar plot illustrates the distribution of tumor mutational burden (TMB) levels, measured in mutations per megabase, across various tumor entities. The gray bars represent the median TMB value for each tumor type, while the whiskers (antennae) extend to the maximum observed TMB value within each tumor category. The dashed red line represents the cut-off for TMB-high status (10 Mut/Mb).

Fig. S6

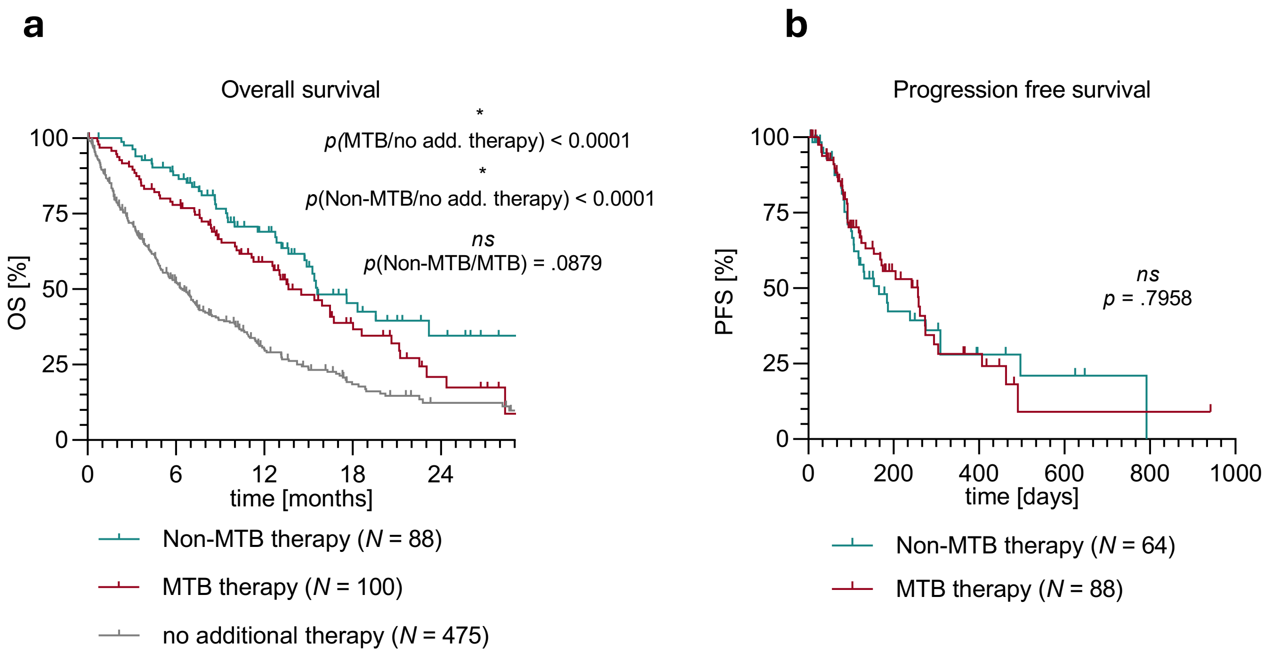

Fig. S6. Survival analyses

(a) Kaplan-Meier survival curves comparing no additional therapy (grey line) vs. MTB Therapy (red line) and Non-MTB therapy (green line) in terms of overall survival (OS) in months. Log-rank was used to assess survival differences between groups as indicated in the figure. The median survival of the MTB therapy group was 13.63 months, of the Non-MTB therapy group 15.60 months and of the no additional therapy group 6.44 months.

(b) Progression-free survival (PFS) curve for patients receiving MTB Therapy (red line, median PFS 257 day) and of the Non-MTB therapy group (green line, median PFS 165 months).
